# Effect of antioxidants on primary open-angle glaucoma: a systematic review and meta-analysis

**DOI:** 10.3389/fphar.2025.1625735

**Published:** 2025-09-05

**Authors:** Jie Bao, Yujia Yu, Guosen Chen, Chen Hu, Cong Zhao, Xiang Li

**Affiliations:** ^1^ Eye School of Chengdu University of Traditional Chinese Medicine, Chengdu, China; ^2^ Hospital of Chengdu University of Traditional Chinese Medicine, Chengdu, China

**Keywords:** antioxidants, primary open-angle glaucoma, normal-tension glaucoma, meta-analysis, randomized controlled trials

## Abstract

**Background/Objectives:**

Primary open-angle glaucoma is a major global cause of vision loss, severely impacting quality of life. Although the need for effective treatments is widely recognized, the efficacy and safety of antioxidants remain uncertain. In this systematic review and meta-analysis, we aimed to evaluate the efficacy and safety of antioxidants in treating patients with primary open-angle glaucoma.

**Methods:**

We reviewed studies from PubMed, Embase, Cochrane Library, and Web of Science published up to 30 November 2024. Eligible studies included adults aged 18–80 years with primary open-angle or normal-tension glaucoma, comparing antioxidant treatment with placebo or comparing a combination of topical treatment and antioxidants with topical treatment alone. Only randomized controlled trials and crossover trials were included. Studies involving secondary glaucoma, ocular inflammation, trauma, or severe systemic disease were excluded, as were nonhuman studies. Of the 518 studies, 15 (2.9%) met the final criteria. Data abstraction and quality assessment followed established guidelines for rigor and transparency. The study outcomes—intraocular pressure, visual field deterioration, ocular blood circulation, blood pressure, and adverse effects—were chosen to evaluate the efficacy and safety of antioxidant treatments in primary open-angle glaucoma.

**Results:**

Analysis of 15 studies showed that antioxidant supplementation reduces intraocular pressure, improves visual field mean deterioration, and enhances ocular blood circulation in patients with primary open-angle glaucoma. No significant differences were observed in blood pressure or adverse effects between the treatment and placebo groups.

**Conclusion:**

This meta-analysis highlights the potential role of antioxidants as a safe and effective therapeutic option for patients with primary open-angle glaucoma.

## 1 Introduction

Glaucoma is a group of diseases characterized by pathological elevation of intraocular pressure (IOP), progressive optic nerve damage, and visual field defects (Stein et al., 2021), making it the leading cause of irreversible blindness worldwide (Steinmetz et al., 2021). By 2040, an estimated 111.8 million individuals aged 40–80 years will be affected globally (Tham et al., 2014). Glaucoma is classified into primary, secondary, and congenital subtypes. Primary glaucoma is further categorized into primary open-angle glaucoma (POAG) and primary angle-closure glaucoma (PACG) based on the anatomical structure of the anterior chamber angle. POAG is characterized by an open anterior chamber angle with increased resistance to aqueous humor outflow through the trabecular meshwork. In contrast, PACG results from angle closure due to iridotrabecular contact, obstructing aqueous outflow (Jayaram et al., 2023). Additionally, optic nerve damage and visual field loss may occur without elevated IOP, a subtype known as normal-tension glaucoma (NTG), which is considered a variant of POAG (Leung and Tham, 2022). POAG is the most prevalent form of glaucoma, accounting for approximately 74%–90% of all cases, predominantly affecting individuals aged >40 years (Quigley and Broman, 2006; Tham et al., 2014; Jin, 2022). POAG is characterized by the progressive loss of retinal ganglion cells (RGCs) and their axons, leading to optic nerve atrophy. The pathogenesis of POAG is multifactorial, with elevated IOP identified as a primary risk factor (Weinreb and Khaw, 2004). Increased IOP induces lamina cribrosa deformation, which disrupts the axonal transport of neurotrophic factors, ultimately causing RGC death due to insufficient trophic support. In addition to IOP, reduced ocular blood flow and localized ischemia at the optic nerve head contribute to chronic ischemic damage and cellular dysfunction (Weinreb et al., 2014; Weinreb and Khaw, 2004). Although IOP control remains fundamental in slowing glaucoma progression (Weinreb and Khaw, 2004; Lusthaus and Goldberg, 2019), some patients experience continued disease progression despite effective IOP management (Almasieh and Levin, 2017; Oliver et al., 2002). This indicates that, beyond IOP regulation, interventional strategies targeting other pathological mechanisms warrant further exploration (Weinreb et al., 2014; Jayaram et al., 2023; Bou Ghanem et al., 2024).

Recent studies have highlighted the pivotal role of oxidative stress (OS) in the pathogenesis and progression of POAG (Evangelho et al., 2019; Kwon et al., 2009). Antioxidants may hold potential value in slowing the progression of POAG by scavenging free radicals and alleviating oxidative damage (Kwon et al., 2009; Boccaccini et al., 2023). Increasing evidence exists that OS levels are elevated in patients with glaucoma (Hondur et al., 2017; Li et al., 2020; Tang et al., 2019). In several randomized controlled trials (RCTs) and observational studies, researchers have investigated the potential benefits of antioxidants, including vitamins, coenzyme Q10, and flavonoids, in POAG management (Sabaner et al., 2021; Shim et al., 2012; Parisi et al., 2014; Ramdas et al., 2018). However, inconsistencies in findings across these studies have made the true efficacy of antioxidants in POAG unclear. Therefore, this study aimed to systematically review the existing literature to assess the therapeutic potential of antioxidants in patients with POAG.

## 2 Methods

### 2.1 Study protocol and registration

In this systematic review, we aimed to evaluate the efficacy of antioxidants versus placebo and antioxidants combined with topical treatment versus topical treatment alone in alleviating symptoms in patients with POAG. The study protocol was registered in PROSPERO (Registration No. CRD42025629951). The study design adhered to the Preferred Reporting Items for Systematic reviews and Meta-Analyses guidelines (Moher et al., 2009) to ensure methodological rigor.

### 2.2 Search strategy and eligibility criteria

A comprehensive literature search was conducted in the PubMed, Embase, Cochrane Library, and Web of Science databases to identify RCTs evaluating antioxidant treatment in patients with POAG, including studies published up to 30 November 2024. The search strategy was tailored to each database, applied without language restrictions, and excluded nonhuman studies. The search terms included “primary open-angle glaucoma,” “normal-tension glaucoma,” “antioxidants,” and “randomized controlled trials.” Specific antioxidant subtypes, such as vitamins, coenzyme Q10, and flavonoids, were also considered (detailed search strategies and keywords are provided in Supplementary Material 1). To enhance the comprehensiveness of this review, the reference lists of relevant studies were screened. All identified studies were managed using EndNote 20. After duplicate removal, titles, abstracts, and full texts were screened and assessed according to predefined eligibility criteria.

### 2.3 Inclusion and exclusion criteria

The inclusion criteria were as follows: (1) Age 18–80 years; (2) Diagnosis of POAG or NTG in at least one eye, confirmed by glaucomatous optic disc or retinal nerve fiber layer abnormalities and/or visual field defects consistent with glaucoma, with the exclusion of other ocular or systemic neurological conditions that could cause visual field defects; (3) Studies comparing antioxidant treatment with placebo or studies comparing a combination of topical treatment and antioxidants with topical treatment alone and (4) RCTs.

The exclusion criteria were as follows: (1) Secondary glaucoma (e.g., pseudoexfoliative or pigmentary glaucoma), angle-closure glaucoma, or congenital glaucoma; (2) History of chronic or recurrent ocular inflammation, ocular trauma, glaucoma surgery (except uncomplicated cataract surgery performed at least 6 months prior), severe or progressive retinal disease, or severe cardiovascular, renal, or pulmonary disease; (3) Pregnancy or lactation; (4) Animal or *in vitro* studies; and (5) Studies that were withdrawn, as well as reviews, meta-analyses, case reports, protocols, conference abstracts, book chapters, and non-English literature.

### 2.4 Data collection and quality assessment

Two independent reviewers screened the titles, abstracts, and full texts to identify eligible studies. Any disagreements were resolved through discussion or, if necessary, consultation with a third reviewer. One reviewer conducted data extraction, which was independently verified by another reviewer. Any discrepancies were addressed through discussion. The extracted data included study characteristics such as the first author, publication year, country, glaucoma type, sample size, sex, age, intervention details, outcomes, and follow-up duration.

The risk of bias was assessed using the Cochrane Risk-of-Bias tool, which evaluates the following seven domains: random sequence generation, allocation concealment, blinding of participants, blinding of outcome assessors, incomplete outcome data, selective reporting, and other biases. Each domain was rated as low, high, or unclear risk, and an overall risk assessment was assigned to each study.

### 2.5 Statistical analysis

The clinical efficacy and safety of antioxidant treatments for POAG were assessed using the following outcome measures: IOP, mean visual field deterioration, ocular blood circulation, blood pressure, and adverse effects. Meta-analyses were performed for IOP, mean visual field deterioration, ocular blood circulation, and blood pressure by analyzing score changes before and after the intervention. Because most studies did not report significant differences in adverse effects between groups, a comprehensive meta-analysis was not feasible; therefore, only a descriptive analysis of adverse effects was conducted. Due to variations in intervention methods across studies, subgroup analyses were performed to compare antioxidant treatment with placebo, as well as the combination of topical treatment and antioxidants with topical treatment alone. Additional subgroup analyses were conducted based on different ocular blood flow measurement sites, including the optic nerve head, superior temporal disc rim, inferior temporal disc rim, superior temporal peripapillary retina, and inferior temporal peripapillary retina.

Data analyses were conducted using Review Manager 5.4. For continuous data measured on the same scale, the mean difference (MD) with 95% confidence intervals (CIs) was calculated. The standardized mean difference (SMD), with 95% CIs, was used when data were reported on different scales. Heterogeneity among studies was assessed using the *I* ^*2*^ statistic, interpreted according to the Cochrane Handbook, with 0%–40%, 30%–60%, 50%–90%, and 75%–100% indicating minimal, moderate, substantial, and considerable heterogeneity, respectively. A fixed-effects model was applied for all analyses. The experimental group comprised participants who received antioxidants alone or in combination with topical treatment, whereas the control group included those who received either a placebo or topical treatment alone. To account for differences in measurement time points across studies, the final recorded time point from each study was used in the analysis.

### 2.6 Quality of evidence

The quality of evidence was assessed using the GRADE scoring system (Balshem et al., 2011). According to GRADE methodology, evidence from RCTs is initially classified as high quality but may be downgraded due to factors such as imprecision, inconsistency, indirectness, or publication bias. The evidence was categorized into the following two subgroups based on treatment type: (1) antioxidant treatment versus placebo and (2) a combination of topical treatment and antioxidants versus topical treatment alone.

## 3 Results

### 3.1 Characteristics of included studies

**FIGURE 1:**
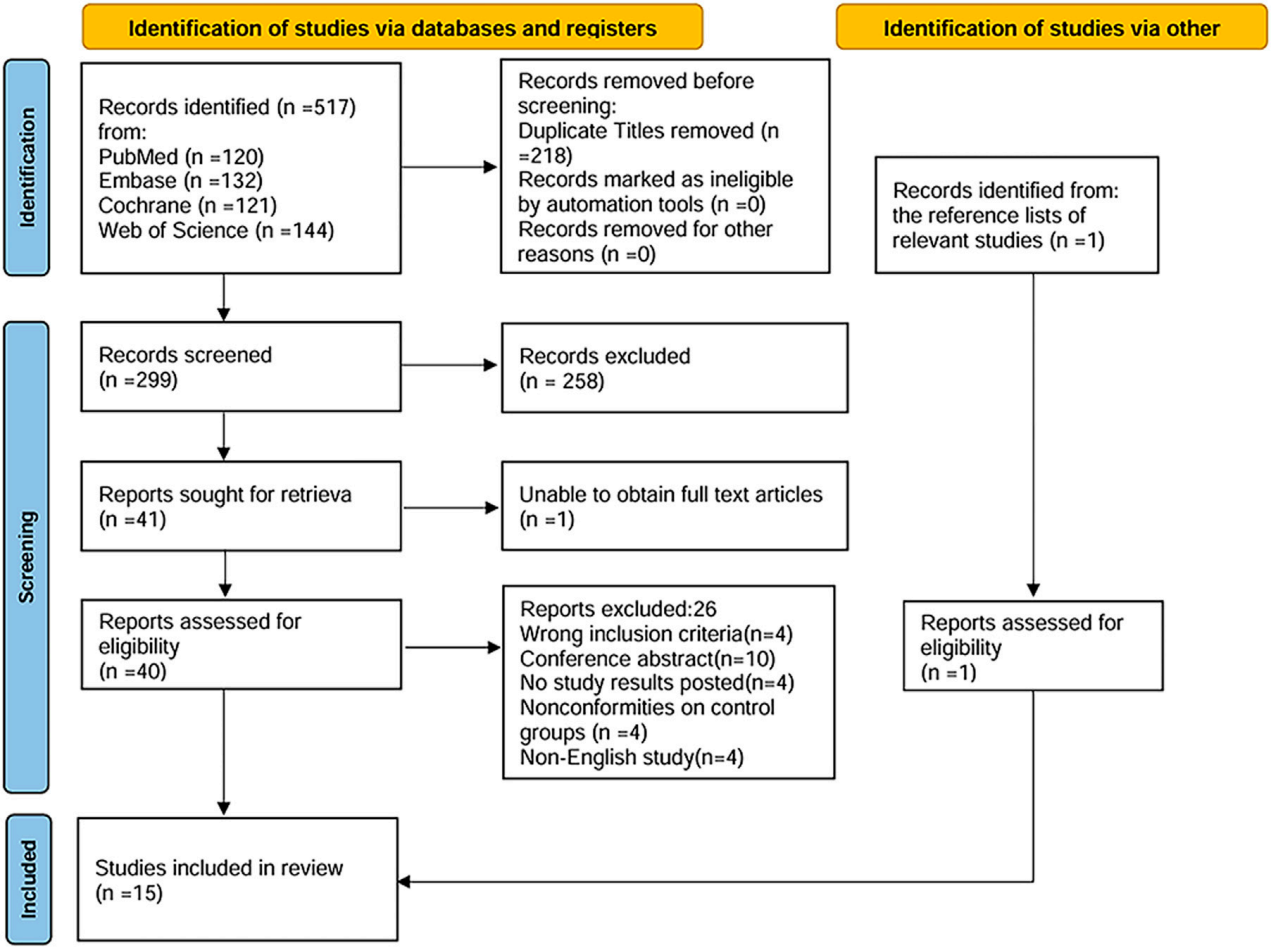
Flow diagram of literature search and selection process according to the Preferred Reporting Items for Systematic reviews and Meta-Analyses statement.

Overall, 518 relevant studies were identified through the search strategy. After removing 218 duplicate articles, 300 articles underwent title and abstract screening. Of these, 258 were excluded due to reviews, nonhuman studies, retrospective studies, protocols, case reports, or failure to meet the inclusion criteria. Following this initial screening, 42 articles proceeded to full-text review. Secondary screening led to the exclusion of several studies: four studies that did not meet the inclusion criteria, 10 conference abstracts, four studies lacking reported outcome data, four studies with inappropriate control groups, four non-English studies, and one study for which the full text was not accessible. Ultimately, 15 studies (Harris et al., 2018; Jabbarpoor Bonyadi et al., 2014; Garcia-Medina et al., 2015; Guo et al., 2014; Hao et al., 2018; Mahdiani et al., 2024; Ohguro et al., 2012; Ohguro et al., 2013; Parisi et al., 2014; Park et al., 2011; Quaranta et al., 2003; Sari et al., 2016; Yoshida et al., 2013; Vetrugno et al., 2012; Zhong et al., 2010) met the inclusion criteria and were included in the meta-analysis. The study screening and selection process is illustrated in Figure 1. The major characteristics of the included studies are summarized in Table 1.

**TABLE 1:** Summary of the included studies.

Study	Study design	Country and participants	Sample description		Intervention		Outcome and duration
			Case	Control	Case	Control	
Harris et al. (2018)	Cross over trial	USA OAG	42 (24/18) 67.1 ± 10.9	42 (24/18) 67.1 ± 10.9	Four soft gels daily of dietary supplement→ washout→ four soft gels daily of placebo	Four soft gels daily of placebo→ washout→ four soft gels daily of dietary supplement	HR, BP, IOP, and ocular blood flow 1 month
Bou Ghanem et al. (2014)	RCT	Iran POAG	17 (10/7) 66.3 ± 9.5	17 (11/6) 67.6 ± 8.3	Topical timolol 0.5% two times daily, and dorzolamide 2% three times daily One saffron capsule daily	Topical timolol 0.5% two times daily, and dorzolamide 2% three times daily One placebo capsule daily	IOP 4 weeks
Garcia-Medina et al. (2015)	RCT	Spain POAG	54 (21/33) 60.76 ± 12.21	63 (28/35) 63.14 ± 10.76	Topical antiglaucoma medications, one antioxidant-containing capsule after breakfast from Monday to Friday	Topical antiglaucoma medications	VF, RNFL parameters, and GCC parameters 2 years
Guo et al. (2014)	Cross over trial	China NTG	28 (16/12) 63.7 ± 6.5	28 (16/12) 63.7 ± 6.5	A capsule containing 40 mg of ginkgo biloba three times a day→washout→Identical placebo capsules three times daily	Identical placebo capsules three times daily→washout→a capsule containing 40 mg of ginkgo biloba three times a day	VF, IOP, BP, contrast sensitivity, and side effects 4 weeks
Hao et al. (2018)	RCT	China POAG	58 (28/30) 58.3 ± 12.3	50 (26/24) 58.3 ± 12.3	Artificial tear solution, Puerarin 2 mg/kg	Artificial tear solution, placebo 2 mg/kg	IOP, VA, TBUT, VF defect, inflammation score, corneal erosion, subjective discomfort, and side effects 6 months
Mahdiani et al. (2024)	RCT	Iran POAG	20 (11/9) 56.88 ± 11.38	20 (7/13) 55.17 ± 14.6	Topical medications: 15 mg crocin tablet every day	Topical medications, identical placebo tablet every day	IOP, BCVA, CDR, and RNFL thickness 4 months
Ohguro (2012)	RCT	Japan OAG	20 (13/7) 60.35 ± 7.22	20 (10/10) 63.20 ± 14.78	Take two capsules containing BCAC once a day	Take two placebo capsules once a day	IOP, VF, BP, PR, and ocular blood flow 2 years
Ohguro et al. (2013)	RCT	Japan OAG	12 (8/4) 61.42 ± 6.95	9 (6/3) 64.78 ± 14.39	Prostaglandin analogs, two capsules containing BCAC once a day	Prostaglandin analogs, two capsules containing a placebo once a day	IOP, VF, BP, and PR 2 years
Parisi et al. (2014)	RCT	Spain OAG	22 52.8 ± 5.46	21 52.1 ± 5.22	β-blocker monotherapy, coenzyme Q10, and vitamin E	β-blocker monotherapy	PERG, VEP, and IOP 12 months
Park et al. (2011)	RCT	Korea NTG	15 (7/8) 18–80	15 (4/11) 18–80	80 mg GBE orally two times a day	Placebo orally two times a day	IOP, VF, and ocular blood flow 4 weeks
Quaranta et al. (2003)	Cross over trial	Italy NTG	27 (16/11) 70.4 ± 6.5	27 (16/11) 70.4 ± 6.5	A capsule containing 40 mg of ginkgo biloba three times a day→washout→Identical placebo capsules three times daily	Identical placebo capsules three times daily→washout→a capsule containing 40 mg of ginkgo biloba three times a day	VF and side effects 4 weeks
Sari et al. (2016)	RCT	Indonesia POAG	20 (6/14) 54.63 ± 4.34	20 (8/12) 54.92 ± 4.26	40 mg GBE two times daily	Identical placebo two times daily	IOP, VF, RNFL thickness, CDR, and OS marker 6 months
Yoshida et al. (2013)	RCT	Japan OAG	19 (NA)	19 (NA)	Take two capsules containing BCAC once a day	Take two placebo capsules once a day	Levels of ET-1 concentration, NO, AOPP and antioxidant activities 2 years
Vetrugno et al. (2012)	RCT	Italy POAG	52 (25/27) 63.5 ± 7.62	45 (23/22) 67.4 ± 6.37	Antiglaucoma medication, one supplement tablet two times a day	Antiglaucoma medication	IOP 3 weeks
Zhong et al. (2010)	RCT	China POAG	20 (10/10) 63.00 ± 14.45	20 (11/9) 64.83 ± 13.03	Two tablets containing 40 mg of flavonoids orally three times a day	Two placebo tablets orally three times a day	IOP, CDR, VF, BP, PR and VA 6 months

RCT, randomized control trial; OAG, open-angle glaucoma; POAG, primary open-angle glaucoma; NTG, normal tension glaucoma; IOP, intraocular pressure; VF, visual field; RNFL, retinal nerve fiber layer; GCC, ganglion cell complex; VA, visual acuity; BCVA, best-corrected visual acuity; CDR, cup-to-disc ratio; PERG, pattern electroretinogram; VEP, visual-evoked potential; TBUT, tear film break-up time; NO, nitric oxide; AOPP, advanced oxidation protein products; OS, oxidative stress; BP, blood pressure; HR, heart rate; PR, pulse rate; NA, not applicable; Sample description: sample size (male/female); age (mean ± SD, or median [minimum–maximum]).

### 3.2 Risk of bias

**FIGURE 2:**
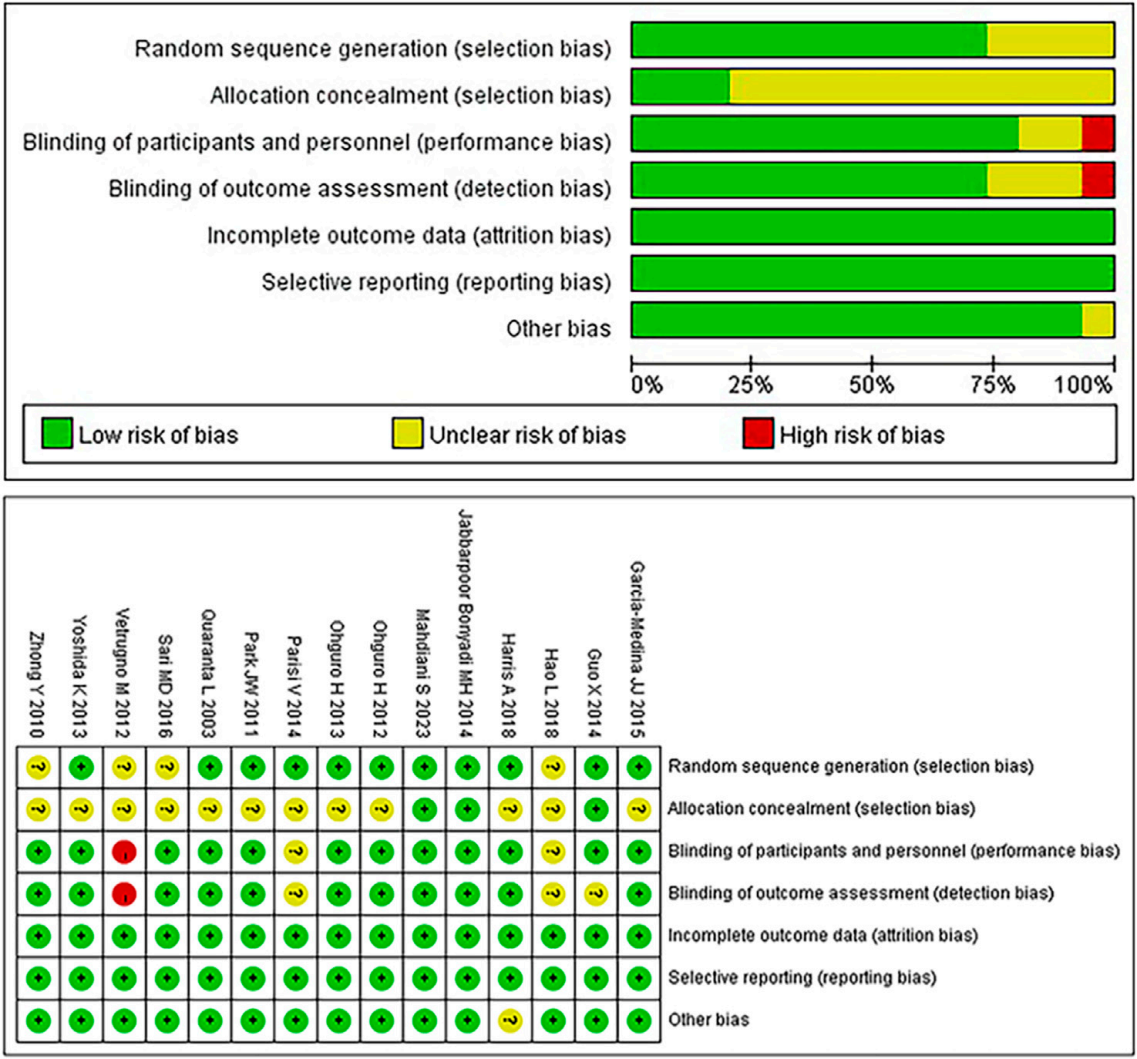
Bias risk of the included studies (Cochrane Collaboration tool).

The risk of bias in the 15 included RCTs was assessed using the Cochrane Risk-of-Bias tool (Higgins et al., 2011); the results are summarized in Figure 2. Two studies met all assessment criteria and were classified as having a low risk of bias. Twelve studies exhibited an unclear risk due to uncertainties in one or more domains. One study was deemed to have a high risk of bias due to the lack of blinding in a specific domain.

### 3.3 Meta-analysis

The meta-analysis included 15 studies, and the results for each outcome measure are detailed below.

#### 3.3.1 Intraocular pressure

**FIGURE 3:**
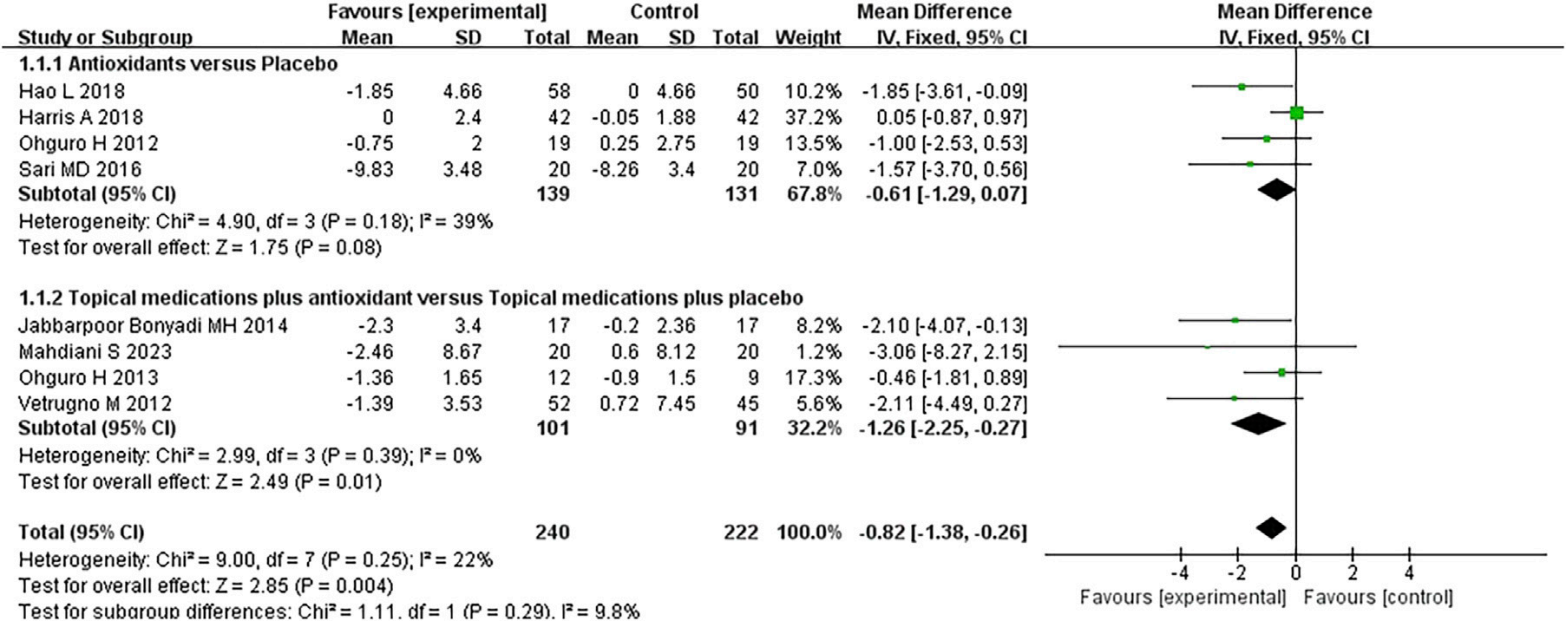
Meta-analysis plot of intraocular pressure (mmHg) in the experimental and control groups.

Eight studies assessing the effect of antioxidants on IOP in patients with POAG were included in the analysis. The overall heterogeneity among the studies was 22%. Subgroup analysis revealed that, compared to placebo, antioxidant treatment did not result in a statistically significant reduction in IOP (*MD* -0.61 [−1.29, 0.07]; *P* = 0.08; *I* ^*2*^ = 39%; *P*_*I*_^*2*^ = 0.18). However, the combination of topical treatment and antioxidants was associated with a significantly greater reduction in IOP than in topical treatment alone (*MD* -1.26 [−2.25, −0.27], *P* = 0.01, *I* ^*2*^ = 0%, *P*_*I*_^*2*^ = 0.39). The overall pooled analysis indicated that the experimental group (n = 240) had a significantly lower IOP than the control group (n = 222) (*MD* -0.82 [−1.38, −0.26], *P* = 0.004; *I* ^*2*^ = 22%, *P*_*I*_^*2*^ = 0.25; Figure 3). Notably, sensitivity analysis excluding one study (Vetrugno et al., 2012) with high risk of bias demonstrated consistent results (*MD* -0.74 [−1.32, −0.16], *P* = 0.01; *I* ^*2*^ = 23%, *P*_*I*_^*2*^ = 0.25), suggesting relative robustness of the findings.

#### 3.3.2 Visual field

**FIGURE 4:**
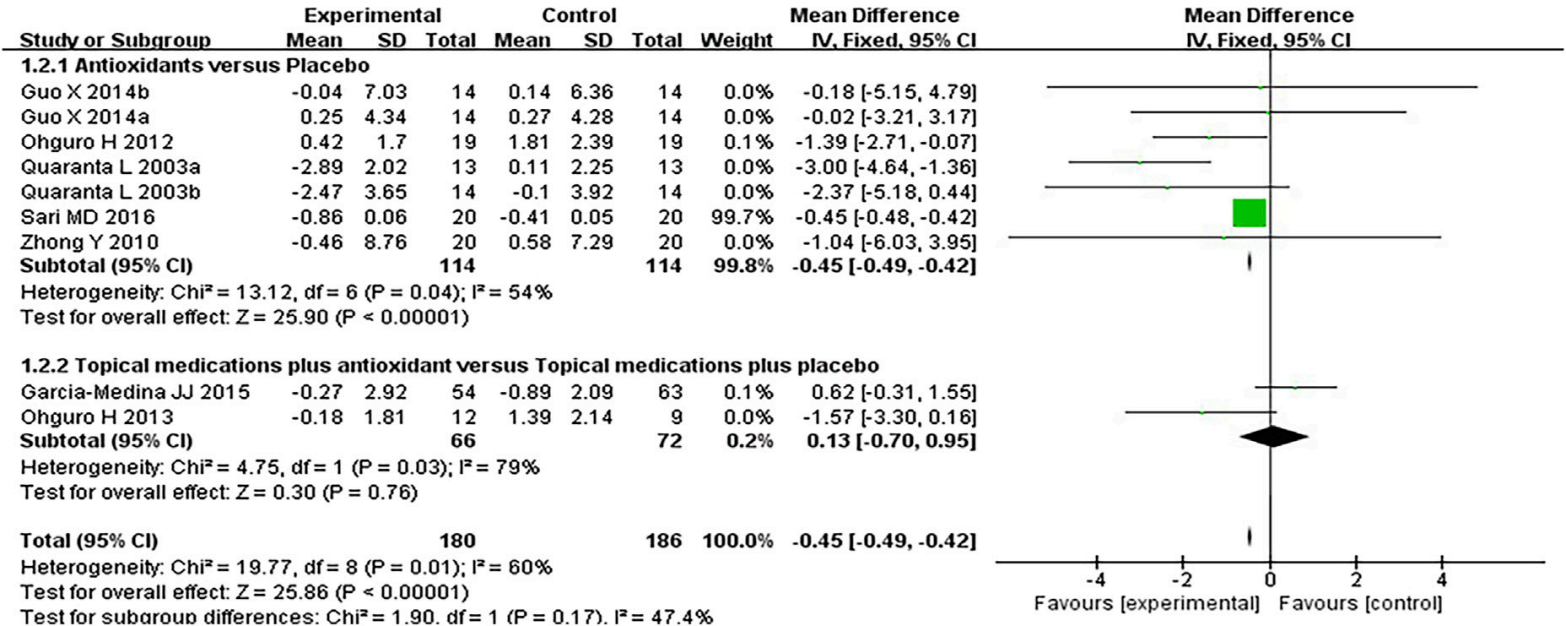
Meta-analysis plot of visual field mean deterioration (dB) in the experimental and control groups.

**FIGURE 5:**
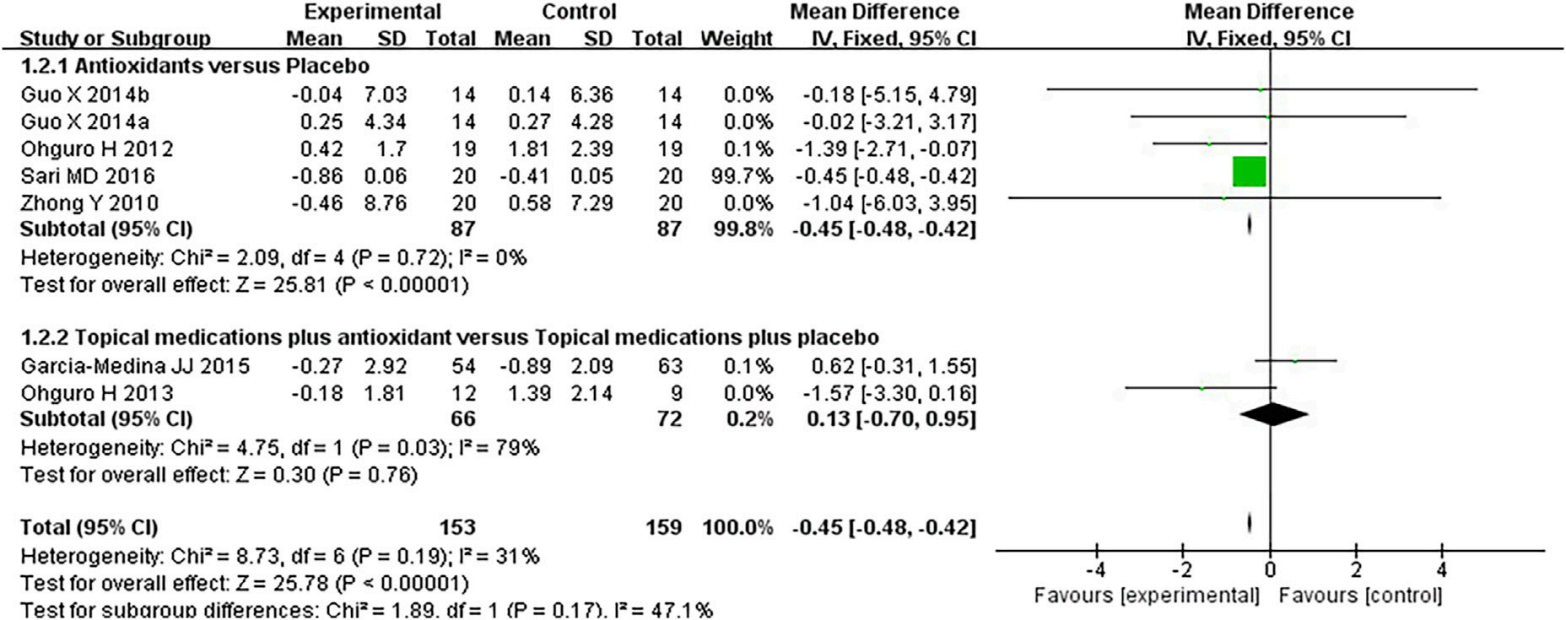
Meta-analysis plot of visual field mean deterioration (dB) in the experimental and control groups.

Seven studies assessed the visual field in patients with POAG. When analyzing the first and second phases of the crossover trial separately, a meta-analysis of the mean visual field deterioration (endpoint value minus baseline value) indicated a statistically significant improvement in the experimental group compared to the control group (*MD* −0.45 [−0.49, −0.42], *P* < 0.00001). Subgroup analysis revealed that antioxidants alone were more effective than placebo (*MD* −0.45 [−0.49, −0.42], *P* < 0.00001, *I* ^*2*^ = 54%, *P*_*I*_^*2*^ = 0.04), whereas combined topical treatment with antioxidants showed no significant difference compared to topical treatment alone (*MD* −0.13 [−0.70, 0.95], *P* = 0.76, *I* ^*2*^ = 79%, *P*_*I*_^*2*^ = 0.03). The overall heterogeneity in the effect of antioxidants on mean visual field deterioration across all studies was moderate (*I* ^*2*^ = 60%, *P*_*I*_^*2*^ = 0.01; Figure 4). Further investigation of this heterogeneity identified the study by Quaranta et al. (2003), where the authors reported results as mean ±2 *SD*, as a potential source of substantial heterogeneity. Excluding this study reduced the heterogeneity to 31% (*MD* -0.45 [−0.48, −0.42], *P* < 0.00001, *I* ^*2*^ = 31%, *P*_*I*_^*2*^ = 0.19; Figure 5).

#### 3.3.3 Ocular blood circulation

**FIGURE 6:**
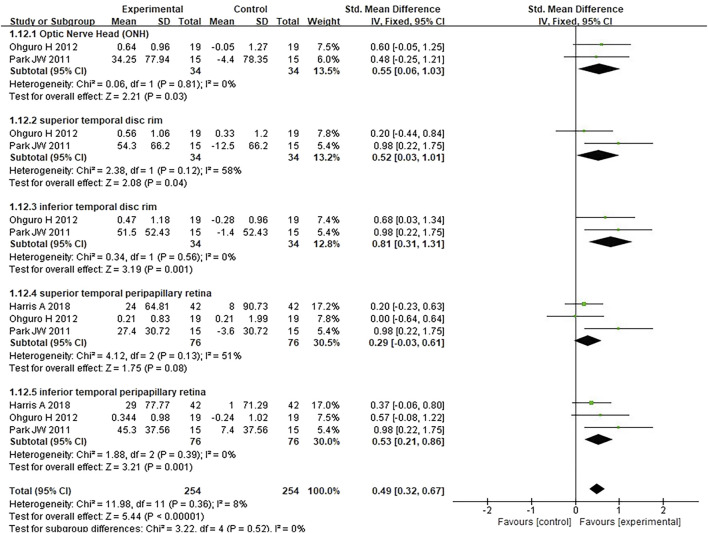
Meta-analysis plot of ocular blood circulation in the experimental and control groups.

Three studies assessing the effect of antioxidants on retinal blood flow in patients with POAG were analyzed; the studies together comprised 12 subgroups, and the measured parameters were as follows: optic nerve head, superior temporal disc rim, inferior temporal disc rim, superior temporal peripapillary retina, and inferior temporal peripapillary retina. Compared to placebo, antioxidants significantly improved blood flow in the optic nerve head (*SMD* 0.55 [0.06, 1.03], *P* = 0.03, *I* ^*2*^ = 0%, *P*_*I*_^*2*^ = 0.81), superior temporal disc rim (*SMD* 0.52 [0.03, 1.01], *P* = 0.04, *I* ^*2*^ = 58%, *P*_*I*_^*2*^ = 0.12), inferior temporal disc rim (*SMD* 0.81 [0.31, 1.31], *P* = 0.001, *I* ^*2*^ = 0%, *P*_*I*_^*2*^ = 0.56), and inferior temporal peripapillary retina (*SMD* 0.53 [0.21, 0.86], *P* = 0.001, *I* ^*2*^ = 0%, *P*_*I*_^*2*^ = 0.39), except for the superior temporal peripapillary retina (*SMD* 0.29 [-0.03, 0.61], *P* = 0.08, *I* ^*2*^ = 51%, *P*_*I*_^*2*^ = 0.13). The pooled analysis indicated that the experimental group (n = 254) exhibited significantly higher retinal blood flow than the control group (n = 254) (*SMD* 0.49 [0.32, 0.67]; *P* < 0.00001; *I* ^*2*^ = 8%; *P*_*I*_^*2*^ = 0.36; Figure 6).

#### 3.3.4 Blood pressure

**FIGURE 7:**
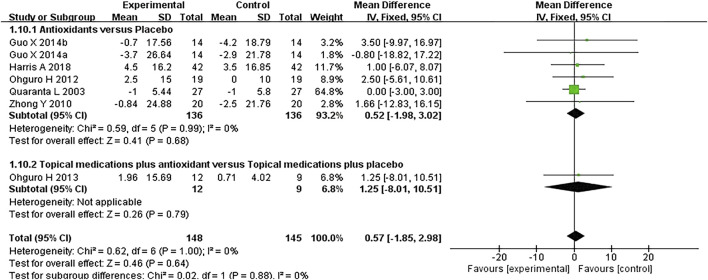
Meta-analysis plot of systolic blood pressure (mmHg) in the experimental and control groups.

**FIGURE 8:**
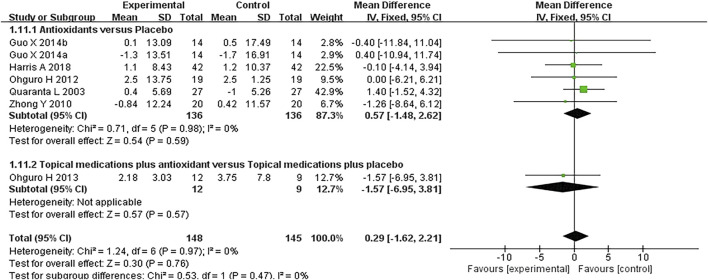
Meta-analysis plot of diastolic blood pressure (mmHg) in the experimental and control groups.

Six studies where systolic and diastolic blood pressure data were reported were included in the analysis, with separate assessments conducted for the first and second phases of the crossover trials. Seven subgroups were included in this meta-analysis. The forest plots for systolic and diastolic blood pressure indicated no statistically significant differences between the experimental and control groups for either systolic blood pressure (*MD* 0.57 [−1.85, 2.98], *P* = 0.64, *I* ^*2*^ = 0%, *P*_*I*_^*2*^ = 1.00) or diastolic blood pressure (*MD* 0.29 [−1.62, 2.21], *P* = 0.76, *I* ^*2*^ = 0%, *P*_*I*_^*2*^ = 0.97) (Figures 7, 8).

### 3.4 Adverse effects

In seven of the studies, the adverse effects of treatment were assessed. Guo et al. (2014) identified two drug-related adverse events during the placebo phase, whereas Mahdiani et al. (2024) reported nine and seven cases during the antioxidant and placebo phases, respectively. No adverse events occurred during treatment in the other studies. No statistically significant differences were observed in adverse effects between the treatment and placebo groups. Adverse events reported by the studies are presented in Table 2.

**TABLE 2:** Adverse events reported by the studies.

Study	Intervention	Adverse events (test Group)	Adverse events (control Group)	Causality assessment
Harris et al. (2018)	4 capsule of antioxidant supplement per day	None	None	—
Jabbarpoor Bonyadi et al. (2014)	Saffron extract 30 mg/day	None	None	—
Garcia-Medina et al. (2015)	1 capsule of antioxidant supplement per day	None	None	—
Guo et al. (2014)	Ginkgo biloba 120 mg/day	None	Gastric discomfort (n = 1), urticaria (n = 1)	Possibly unrelated
Hao et al. (2018)	Puerarin 2 mg/kg	None	None	—
Mahdiani et al. (2024)	Crocin 15 mg/day	Epiphora (n = 2); Increased IOP (n = 1); Nausea (n = 1); Shortness of breath (n = 1); Increased heart rate (n = 1); Red light sensitivity (n = 1); Reduced visibility (n = 2)	Floaters and brown spots in the eye (n = 2); Burning eyes (n = 1); Increased IOP (n = 3); Reduced visibility (n = 1)	Possibly related
Ohguro et al. (2012)	Black currant anthocyanins 50 mg/day	None	None	—
Ohguro et al. (2013)	Black currant anthocyanins 50 mg/day	not reported	not reported	—
Park et al. (2011)	Ginkgo biloba 160 mg/day	None	None	—
Quaranta et al. (2003)	Ginkgo biloba 120 mg/day	None	None	—
Sari et al. (2016)	Ginkgo biloba 80 mg/day	not reported	not reported	—
Yoshida et al. (2013)	Black currant anthocyanins 50 mg/day	not reported	not reported	—
Vetrugno et al. (2012)	Forskolin 30 mg + rutin 400 mg	not reported	not reported	—
Zhong et al. (2010) ^a^	Erigeron breviscapus 240 mg/day	None	None	—
Parisi et al. (2014)	Topical eye drops containing Coenzyme Q10 (100 mg) and Vitamin E TPGS (500 mg), 2drops/day	None	None	—

^a^ Study included in this review used a dose higher than the recommended daily amount.

Across the 15 included studies, Guo et al. (2014) identified two drug-related adverse events (AEs) during the placebo phase, whereas Mahdiani et al. (2024) reported nine and seven AEs during the antioxidant and placebo phases, respectively. Nine studies reported no occurrence of AEs during treatment, while four studies did not document treatment-emergent AEs. No statistically significant differences were observed in AEs between the treatment and placebo groups. AEs reported by the studies are presented in Table 2. Notably, in the study by Zhong et al., the administered metabolite dosage exceeded the recommended daily allowance; however, no AEs were observed in any participant throughout the 6-month trial period.

### 3.5 Level of evidence

**FIGURE 9:**
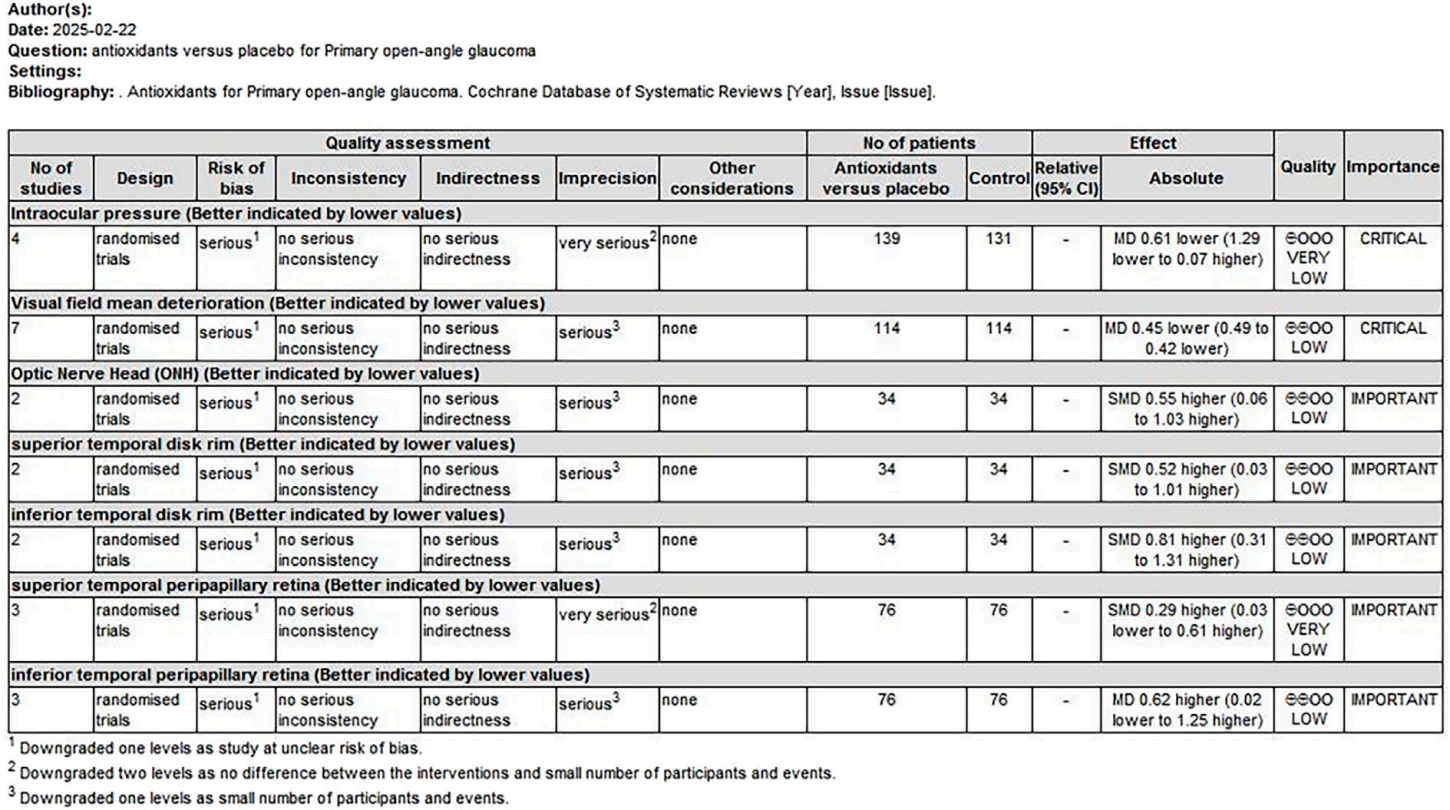
Antioxidant treatment compared to placebo for treating POAG. POAG, primary open-angle glaucoma.

**FIGURE 10:**
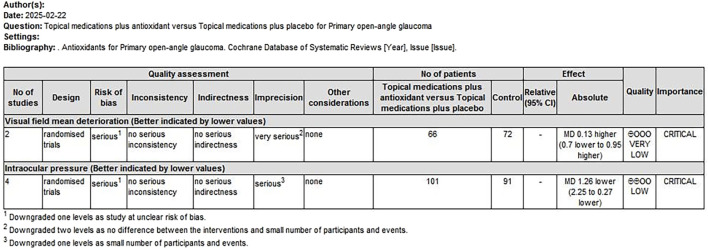
Combination of topical treatment with antioxidants compared to topical treatment alone for treating POAG. POAG, primary open-angle glaucoma.

The quality of evidence was assessed using the GRADE approach. Two overall assessments were performed as follows: (1) a comparison of antioxidants with placebo (Figure 9) and (2) a comparison of combined topical treatment with antioxidants versus topical treatment alone (Figure 10). The overall quality of evidence ranged from “very low” to “low.” The primary factors contributing to evidence downgrading were the risk of bias and the small sample size.

## 4 Discussion

Antioxidants have been widely studied for their potential to enhance ocular health in patients with glaucoma; however, their specific effects on POAG have not been systematically evaluated. The results of our meta-analysis indicate that antioxidant treatment significantly reduces IOP in patients with POAG and leads to statistically significant improvements in mean visual field deterioration and ocular blood circulation. Conversely, we found no significant differences in blood pressure or adverse effects between antioxidant and placebo treatments. The quality of evidence supporting significant outcome differences, as assessed using the GRADE scoring system, ranged from grade D to C for both comparisons: antioxidants versus placebo and antioxidants combined with topical treatment versus topical treatment alone. The strength of evidence was rated at level 2 allocation concealment methods or blinding were not indicated in all of the studies, and the overall quality of the evidence was limited due to inadequate study design and small sample sizes, contributing to the downgrading of evidence levels. These preliminary findings suggest that antioxidant supplementation may offer adjunctive benefits in POAG. However, the low certainty of evidence (GRADE: D to C) underscores the need for large-scale, rigorously designed RCTs to validate these effects before clinical recommendations can be established.

OS is broadly defined as an imbalance between the production of reactive oxygen species (ROS) and the efficacy of antioxidant defense mechanisms. This imbalance results in damage to macromolecules such as DNA, proteins, and lipids, ultimately triggering apoptosis. OS is a common pathological mechanism underlying various neurodegenerative diseases, including glaucoma (Nita and Grzybowski, 2016; Tezel, 2006; Aruoma, 1998; Andersen, 2004). There is increasing evidence from both animal models and clinical studies that OS is present in the ocular tissues of experimental glaucoma models and patients with clinical glaucoma (Jassim et al., 2021; Benoist d'Azy et al., 2016). OS plays a pivotal role in the progression of glaucoma through a dual mechanism. Firstly, OS directly disrupts the structure and function of the trabecular meshwork, obstructing aqueous humor outflow and thereby contributing to pathological elevation of IOP (Izzotti et al., 2006; Vernazza et al., 2020; Chrysostomou et al., 2013). Secondly, OS-induced vascular alterations compromise the autoregulation of optic nerve blood flow, leading to localized ischemia (Izzotti et al., 2006; Raaz et al., 2014; Chrysostomou et al., 2013). These mechanisms act synergistically: elevated IOP exacerbates compression of the optic nerve microvasculature, intensifying ischemia, while ischemia promotes ROS accumulation, further amplifying oxidative stress. This self-perpetuating cycle collectively accelerates retinal ganglion cell (RGC) degeneration.

IOP control has long been the foundation of POAG management. Studies linking OS to glaucoma pathogenesis further indicate that antioxidants could mitigate neuronal degeneration and slow disease progression. By scavenging free radicals, antioxidants can reduce OS, enhance trabecular meshwork function, facilitate aqueous humor outflow, and lower IOP. This reduction in IOP can, in turn, minimize RGC damage, inhibit optic nerve atrophy, and preserve visual field function. Additionally, studies have indicated that antioxidants, including vitamin E and coenzyme Q10, can modulate the ocular redox state, influencing aqueous humor dynamics (Martucci and Nucci, 2019; Engin et al., 2007; Ozates et al., 2019). Antioxidants may also enhance retinal blood flow by improving ocular microcirculation and reducing local ischemic damage. By inhibiting ROS production and enhancement of vascular endothelial function, antioxidants promote ocular blood circulation, optimize blood supply to the optic nerve, and mitigate ischemic injury (Jabbehdari et al., 2021). These findings indicate that antioxidants exert multifaceted protective effects in POAG management by improving IOP regulation, preserving visual field function, and enhancing ocular blood circulation, ultimately contributing to delayed disease progression.

Although antioxidants have shown promising potential in significantly reducing IOP, improving mean visual field deterioration, and enhancing ocular blood circulation, their integration into clinical practice requires caution. First, IOP control remains the cornerstone of glaucoma management. While observed reductions in IOP may reach statistical significance, current guidelines indicate that a clinically meaningful decrease generally requires a ≥20% reduction from baseline (Gedde et al., 2021; Augusto et al., 2021). Consequently, antioxidant monotherapy may have limited efficacy, and combination therapy with conventional IOP-lowering medications may provide additional benefits.

A major limitation of the current evidence is the considerable heterogeneity in antioxidant interventions across the included trials, with substantial variations observed in antioxidant types and inconsistent reporting of metabolite preparation details, dosage regimens, and administration routes in original studies (see Table 3) Additionally, some studies evaluated antioxidants as adjuncts to conventional IOP-lowering therapies, whereas others administered them as monotherapy. These clinical and methodological variations make it challenging to determine whether the observed effects can be attributed to any specific metabolite, dosage, or regimen. Consequently, the pooled results should be interpreted as reflecting the average effect of antioxidants as a class, rather than the precise efficacy of a particular preparation. This heterogeneity also limits the generalizability of the findings, especially in clinical practice, where treatment decisions require drug-specific evidence regarding optimal dosage and duration. Future research should therefore focus on rigorously designed RCTs that directly compare different antioxidants using standardized dosages, uniform treatment courses, and objective biological endpoints to identify potentially superior metabolites or regimens, thereby providing more precise evidence for clinical application. Methodologically, several studies lacked adequate reporting of key design elements, including randomization procedures, allocation concealment, and blinding methods, which may introduce bias and undermine the internal validity of the results. Future clinical trials should rigorously adhere to the CONSORT guidelines to improve methodological quality and ensure transparent reporting of critical trial metabolites.

**TABLE 3:** Detailed information on botanical drug/antioxidant preparations in included studies.

Study	Botanical drug/Antioxidant	Taxonomic validation (POWO/MPNS)	Pharmacopeial standard	Reported composition	Complete details in original study?
Harris et al. (2018)	Antioxidant blend	N/A (non-botanical)	—	Blend of ingredients	Yes (full composition listed)
Jabbarpoor Bonyadi et al. (2014)	Saffron extract	Crocus sativus L. [Iridaceae] (POWO: urn:lsid:ipni.org:names:436688-1)	Crocus sativus L., stigmas (Ph. Eur. 11)	30 mg saffron stigma extract	Partial (no active compound quantification)
Garcia-Medina et al. (2015)	Synthetic antioxidants	N/A (non-botanical)	—	Blend of ingredients	Yes (full composition listed)
Guo et al. (2014)	Ginkgo leaf extract	Ginkgo biloba L. [Ginkgoaceae] (POWO: urn:lsid:ipni.org:names:262125-1)	Ginkgo Folium (Ph. Eur. 11)	40 mg extract (24% flavonol glycosides, 6% terpene lactones)	Yes (EGb761 standard)
Hao et al. (2018)	Puerarin	Pueraria montana var. Lobata (Willd.) (POWO: urn:lsid:ipni.org:names:967441-1)	None	Puerarin 2 mg/kg (≥98% purity)	Yes (purity specified)
Mahdiani et al. (2024)	Crocin	Crocus sativus L. [Iridaceae] (POWO: urn:lsid:ipni.org:names:436688-1)	Crocus sativus L., stigmas (Ph. Eur. 11)	15 mg crocin (≥90% purity by HPLC)	Yes (analytical validation)
Ohguro et al. (2012)	Black currant anthocyanins	Ribes nigrum L. [Grossulariaceae] (POWO: urn:lsid:ipni.org:names:792873-1)	None	50 mg anthocyanins	Yes (doses specified)
Ohguro et al. (2013)	Black currant anthocyanins	Ribes nigrum L. [Grossulariaceae] (POWO: urn:lsid:ipni.org:names:792873-1)	None	50 mg anthocyanins	Yes (doses specified)
Park et al. (2011)	Ginkgo leaf extract	Ginkgo biloba L. [Ginkgoaceae] (POWO: urn:lsid:ipni.org:names:262125-1)	Ginkgo Folium (Ph. Eur. 11)	80 mg extract (19.2 mg flavonol glycosides)	Yes (standardized extract)
Quaranta et al. (2003)	Ginkgo leaf extract	Ginkgo biloba L. [Ginkgoaceae] (POWO: urn:lsid:ipni.org:names:262125-1)	Ginkgo Folium (Ph. Eur. 11)	40 mg extract (24% flavonol glycosides, 6% terpene lactones)	Yes (EGb761 standard)
Sari et al. (2016)	Ginkgo leaf extract	Ginkgo biloba L. [Ginkgoaceae] (POWO: urn:lsid:ipni.org:names:262125-1)	Ginkgo Folium (Ph. Eur. 11)	40 mg extract (24% flavonol glycosides, 6% terpene lactones)	Yes (EGb761 standard)
Yoshida et al. (2013)	Black currant anthocyanins	Ribes nigrum L. [Grossulariaceae] (POWO: urn:lsid:ipni.org:names:792873-1)	None	50 mg anthocyanins	Yes (doses specified)
Vetrugno et al. (2012)	Forskolin + rutin	Coleus hadiensis (Forssk.) A.J.Paton [Lamiaceae] (POWO: urn:lsid:ipni.org:names:77201104-1) + Styphnolobium japonicum (L.) Schott [Fabaceae] (POWO: urn:lsid:ipni.org:names:1119529-2)	None	Forskolin 15 mg + rutin 200 mg	Yes (ratios provided)
Zhong et al. (2010)	Erigeron breviscapus extract	Erigeron breviscapus (Vaniot) Hand.-Mazz. [Asteraceae] (POWO: urn:lsid:ipni.org:names:203633-1)	None	40 mg scutellarin (≥85% purity)	Yes (pharmacopeial standard)
Parisi et al. (2014)	Coenzyme Q10 + Vitamin E	N/A (synthetic)	—	Topical eye drops containing Coenzyme Q10 (100 mg) and Vitamin E TPGS (500 mg)	Yes (doses specified)

## 5 Conclusion

Antioxidants significantly reduce IOP, slow visual field deterioration, and enhance ocular blood circulation in patients with POAG. These findings indicate that antioxidants could be an effective adjunct therapy for POAG management. However, current evidence requires further validation through large-scale, high-quality RCTs to ensure the robustness and reliability of these findings. Furthermore, future studies should incorporate standardized functional and structural assessment metrics to more comprehensively evaluate the clinical utility of antioxidants in the management of POAG.

## Data Availability

The original contributions presented in the study are included in the article/Supplementary Material, further inquiries can be directed to the corresponding author.

## Abbreviations

IOP, intraocular pressure; POAG, primary open-angle glaucoma; PACG, primary angle-closure glaucoma; NTG, normal-tension glaucoma; RGCs, retinal ganglion cells; OS, oxidative stress; RCTs, randomized controlled trials; MD, mean difference; CIs, confidence intervals; SMD, standardized mean difference; ROS, reactive oxygen species.
