# Supplementary material for: Effect of antioxidants on primary open-angle glaucoma: a systematic review and meta-analysis
Source: Front Pharmacol. 2025 Sep 5;16:1625735. doi: 10.3389/fphar.2025.1625735 (PMC12446234; doi:10.3389/fphar.2025.1625735)
Supplement: Supplementary file 1 [file Table1.docx]

**Supplementary Material**:

Table S1. Search strategy on each electronic database

| **Database** | **Search format** |
| --- | --- |
| PubMed | (((Glaucoma, Open-Angle[MeSH Terms]) OR ("Open-Angle Glaucomas"[Title/Abstract] OR "Glaucoma Simplex"[Title/Abstract] OR "Simplex, Glaucoma"[Title/Abstract] OR "Glaucoma, Compensated"[Title/Abstract] OR "Compensated Glaucoma"[Title/Abstract] OR "Glaucoma, Open Angle"[Title/Abstract] OR "Open Angle Glaucoma"[Title/Abstract] OR "Open Angle Glaucomas"[Title/Abstract] OR "Glaucoma, Pigmentary"[Title/Abstract] OR "Pigmentary Glaucoma"[Title/Abstract] OR "Glaucoma, Simple"[Title/Abstract] OR "Simple Glaucoma"[Title/Abstract] OR "Open-Angle Glaucoma"[Title/Abstract] OR "Glaucoma, Primary Open Angle"[Title/Abstract] OR "Primary Open Angle Glaucoma"[Title/Abstract] OR "Chronic Primary Open Angle Glaucoma"[Title/Abstract] OR "Secondary Open Angle Glaucoma"[Title/Abstract] OR "Glaucoma, Secondary Open Angle"[Title/Abstract])) OR (("Low Tension Glaucoma"[MeSH Terms]) OR ("Glaucoma, Low Tension"[Title/Abstract] OR "Low Tension Glaucomas"[Title/Abstract] OR "Normal Tension Glaucoma"[Title/Abstract] OR "Glaucoma, Normal Tension"[Title/Abstract] OR "Normal Tension Glaucomas"[Title/Abstract]))) AND ((((((((((((((((((((((antioxidants[MeSH Terms]) OR (Anti-Oxidant[Title/Abstract] OR "Anti Oxidant"[Title/Abstract] OR Anti-Oxidants[Title/Abstract] OR "Anti Oxidants"[Title/Abstract] OR Antioxidant[Title/Abstract] OR "Antioxidant Effect"[Title/Abstract] OR "Antioxidant Effects"[Title/Abstract] OR "Anti-Oxidant Effect"[Title/Abstract] OR "Anti Oxidant Effect"[Title/Abstract] OR "Anti-Oxidant Effects"[Title/Abstract] OR "Anti Oxidant Effects"[Title/Abstract] OR "Antioxidant Activity"[Title/Abstract] OR "Activity, Antioxidant"[Title/Abstract] OR "Endogenous Antioxidants"[Title/Abstract] OR "Antioxidants, Endogenous"[Title/Abstract] OR "Endogenous Antioxidant"[Title/Abstract] OR "Antioxidant, Endogenous"[Title/Abstract])) OR ((vitamin a[MeSH Terms]) OR ("Aquasol A"[Title/Abstract] OR Retinol[Title/Abstract] OR "Vitamin A1"[Title/Abstract] OR All-Trans-Retinol[Title/Abstract] OR "All Trans Retinol"[Title/Abstract] OR 11-cis-Retinol[Title/Abstract]))) OR ((ascorbic acid[MeSH Terms]) OR ("Acid, Ascorbic"[Title/Abstract] OR "L-Ascorbic Acid"[Title/Abstract] OR "Acid, L-Ascorbic"[Title/Abstract] OR "L Ascorbic Acid"[Title/Abstract] OR "Vitamin C"[Title/Abstract] OR "Ferrous Ascorbate"[Title/Abstract] OR "Ascorbate, Ferrous"[Title/Abstract] OR "Magnesium Ascorbate"[Title/Abstract] OR "Ascorbate, Magnesium"[Title/Abstract] OR Hybrin[Title/Abstract] OR "Sodium Ascorbate"[Title/Abstract] OR "Ascorbate, Sodium"[Title/Abstract]))) OR ((tocopherols[MeSH Terms]) OR (Tocopherol[Title/Abstract] OR E-ferol[Title/Abstract] OR "Bio E"[Title/Abstract] OR Biosan[Title/Abstract] OR Lasar[Title/Abstract] OR Davitamon[Title/Abstract] OR "Aquasol E"[Title/Abstract] OR E-Mulsin[Title/Abstract] OR "E Mulsin"[Title/Abstract] OR E-Vicotrat[Title/Abstract] OR "E Vicotrat"[Title/Abstract] OR "E Vitamin E"[Title/Abstract] OR Evion[Title/Abstract] OR Ephynal[Title/Abstract] OR Spondyvit[Title/Abstract] OR "Unique E"[Title/Abstract] OR Vita-E[Title/Abstract] OR VitaE[Title/Abstract] OR "Vita E"[Title/Abstract] OR "Vitamin E Suspension"[Title/Abstract] OR Vitazell[Title/Abstract]))) OR ((carotenoids[MeSH Terms]) OR (Carotenoid[Title/Abstract] OR Carotenes[Title/Abstract] OR Carotene[Title/Abstract] OR Tetraterpenes[Title/Abstract]))) OR ((beta carotene[MeSH Terms]) OR ("Carotene, beta"[Title/Abstract] OR beta-Carotene[Title/Abstract] OR Betacarotene[Title/Abstract] OR Vetoron[Title/Abstract] OR Carotaben[Title/Abstract] OR Provatene[Title/Abstract] OR Solatene[Title/Abstract]))) OR ((Lycopene[MeSH Terms]) OR (Prolycopene[Title/Abstract] OR Pro-Lycopene[Title/Abstract] OR "Pro Lycopene"[Title/Abstract] OR LYC-O-MATO[Title/Abstract] OR LYCOMATO[Title/Abstract] OR "LYC O MATO"[Title/Abstract] OR All-trans-Lycopene[Title/Abstract] OR "All trans Lycopene"[Title/Abstract]))) OR ((lutein[MeSH Terms]) OR ("Lutein, gamma"[Title/Abstract] OR "Lutein F"[Title/Abstract] OR "Lutein G"[Title/Abstract] OR ((astaxanthine[MeSH Terms]) OR (astaxanthin[Title/Abstract] OR E-astaxanthin[Title/Abstract]))) OR ((Zeaxanthins[MeSH Terms]) OR (Zeaxanthin[Title/Abstract] OR "Beta-Carotene-3,3'-Diol"[Title/Abstract] OR "Beta Carotene 3,3' Diol"[Title/Abstract] OR "3R,3'R-Zeaxanthin"[Title/Abstract] OR "3R,3'R Zeaxanthin"[Title/Abstract]))) OR ((flavonoids[MeSH Terms]) OR (2-Phenyl-Chromene[Title/Abstract] OR "2 Phenyl Chromene"[Title/Abstract] OR Flavonoid[Title/Abstract] OR 2-Phenyl-Benzopyran[Title/Abstract] OR "2 Phenyl Benzopyran"[Title/Abstract] OR Bioflavonoids[Title/Abstract] OR Bioflavonoid[Title/Abstract]))) OR ((anthocyanins[MeSH Terms]) OR (Anthocyanin[Title/Abstract] OR Anthocyanidins[Title/Abstract] OR Anthocyanidin[Title/Abstract] OR Leucoanthocyanidins[Title/Abstract]))) OR ((tea[MeSH Terms]) OR ("Green Tea"[Title/Abstract] OR "Green Teas"[Title/Abstract] OR "Tea, Green"[Title/Abstract] OR "Teas, Green"[Title/Abstract] OR "Black Tea"[Title/Abstract] OR "Black Teas"[Title/Abstract] OR "Tea, Black"[Title/Abstract] OR "Teas, Black"[Title/Abstract]))) OR ((curcumin[MeSH Terms]) OR ("Curcumin Phytosome"[Title/Abstract] OR Diferuloylmethane[Title/Abstract] OR "Turmeric Yellow"[Title/Abstract] OR "Yellow, Turmeric"[Title/Abstract]))) OR ((Resveratrol[MeSH Terms]) OR ("3,4',5-Stilbenetriol"[Title/Abstract] OR "3,5,4'-Trihydroxystilbene"[Title/Abstract] OR "3,4',5-Trihydroxystilbene"[Title/Abstract] OR trans-Resveratrol[Title/Abstract] OR "trans Resveratrol"[Title/Abstract] OR Resveratrol-3-sulfate[Title/Abstract] OR "Resveratrol 3 sulfate"[Title/Abstract] OR "SRT 501"[Title/Abstract] OR SRT-501[Title/Abstract] OR SRT501[Title/Abstract] OR cis-Resveratrol[Title/Abstract] OR "cis Resveratrol"[Title/Abstract] OR "Resveratrol, (Z)-"[Title/Abstract] OR trans-Resveratrol-3-O-sulfate[Title/Abstract] OR "trans Resveratrol 3 O sulfate"[Title/Abstract]))) OR ((Quercetin[MeSH Terms]) OR ("3,3',4',5,7-Pentahydroxyflavone"[Title/Abstract] OR Dikvertin[Title/Abstract]))) OR ((selenium[MeSH Terms]) OR (Selenium-80[Title/Abstract] OR "Selenium 80"[Title/Abstract]))) OR ((Zinc[MeSH Terms]) OR ("Zinc Isotopes"[Title/Abstract] OR "Zinc Radioisotopes"[Title/Abstract]))) OR ((coenzyme Q10[MeSH Terms]) OR ("Bio-Quinone Q10"[Title/Abstract] OR "ubiquinone 10"[Title/Abstract] OR CoQ10[Title/Abstract] OR ubidecarenone[Title/Abstract] OR "ubiquinone 50"[Title/Abstract] OR "ubiquinone Q10"[Title/Abstract] OR "CoQ 10"[Title/Abstract] OR "co-enzyme Q10"[Title/Abstract] OR ubisemiquinone[Title/Abstract] OR "ubisemiquinone radical"[Title/Abstract] OR Q-ter[Title/Abstract]))) OR ((Ginkgo biloba extract[MeSH Terms]) OR ("Ginkgo leaf extract"[Title/Abstract] OR Tebokan[Title/Abstract] OR Tebonin[Title/Abstract] OR "EGb 761"[Title/Abstract] OR EGb-761[Title/Abstract] OR EGb761[Title/Abstract] OR "GBE 761 ONC"[Title/Abstract] OR Rokan[Title/Abstract] OR Tanakan[Title/Abstract] OR "GBE 761"[Title/Abstract] OR GBE-761[Title/Abstract] OR "Ginkgo biloba extract 761"[Title/Abstract]))) OR (melatonin[MeSH Terms])) OR ((ergothioneine[MeSH Terms]) OR (2-Thiol-L-histidine-betaine[Title/Abstract] OR "2 Thiol L histidine betaine"[Title/Abstract] OR Thioneine[Title/Abstract]))) OR ("alpha-Lipoic Acid"[Title/Abstract] OR "Acid, alpha-Lipoic"[Title/Abstract] OR "alpha Lipoic Acid"[Title/Abstract]))) AND ((randomized controlled trial[pt] OR controlled clinical trial[pt] OR randomized[tiab] OR placebo[tiab] OR drug therapy[sh] OR randomly[tiab] OR trial[tiab] OR groups[tiab]) NOT (animals[mh] NOT humans[mh])) |
| Web of Science | #1 TS=(“low tension glaucoma” OR “Normal Tension Glaucomas” OR “ Normal Tension Glaucoma” OR “ Glaucoma, Normal Tension” OR “ Glaucoma, Low Tension” OR “ Low Tension Glaucomas” OR “Glaucoma, Open-Angle” OR “Glaucoma, Secondary Open Angle” OR “ Secondary Open Angle Glaucoma” OR “ Open-Angle Glaucoma” OR “ Open Angle Glaucoma” OR “ Glaucoma, Pigmentary” OR “ Simplex, Glaucoma” OR “ Compensative Glaucoma” OR “ Pigmentary Glaucoma” OR “ Simple Glaucoma” OR “ Glaucoma, Simple” OR “ Glaucoma, Open Angle” OR “ Open Angle Glaucomas” OR “ Open-Angle Glaucomas” OR “Compensated Glaucoma” OR “ Glaucoma Simplex” OR “Glaucoma, Compensated” OR “ Chronic Primary Open Angle Glaucoma” OR “ Glaucoma, Primary Open Angle” OR “ Primary Open Angle Glaucoma”)  #2 TS=(“Antioxidants” OR “Anti Oxidant” OR “Anti-Oxidants” OR “Anti-Oxidant” OR “Antioxidant” OR “Anti Oxidants” OR “Activity, Antioxidant” OR “Antioxidant Activity” OR “Anti-Oxidant Effects” OR “Anti Oxidant Effect” OR “Antioxidant Effect” OR “Anti Oxidant Effects” OR “Anti-Oxidant Effect” OR “Antioxidant Effects” OR “Antioxidant, Endogenous” OR “Antioxidants, Endogenous” OR “Endogenous Antioxidants” OR “Endogenous Antioxidant” OR “Vitamin A” OR “All Trans Retinol” OR “Retinol” OR “Vitamin A1” OR “quasol A” OR “Ascorbic Acid” OR “Magnorbin” OR “Vitamin C” OR “L Ascorbic Acid” OR “Acid, Ascorbic” OR “Ascorbate, Sodium” OR “Sodium Ascorbate” OR “Ascorbic Acid, Monosodium Salt” OR “Magnesium di L Ascorbate” OR “Magnesium Ascorbate” OR “Magnesium Ascorbicum” OR “Ascorbate, Magnesium” OR “Ferrous Ascorbate” OR “Ascorbate, Ferrous” OR “Hybrin” OR “Tocopherols” OR “Vitamin E, Vitagutt” OR “Vitagutt Vitamin E” OR “Mowivit Vitamin E” OR “Vitamin E, Mowivit” OR “Vitamin E EVI MIRALE” OR “Vitamine E GNR” OR “Vitamin E Dragees” OR “E Vitamin E” OR “Vitamin E mp” OR “Vitamin E Suspension” OR “Vitamin E, Togasan” OR “Togasan Vitamin E” OR “Vitamin E Natur” OR “EUNOVA Vitamin E” OR “Vitamin E AL” OR “Vitamin E Sanum” OR “Carotenoids” OR “Carotenoid” OR “Tetraterpenes” OR “Tetraterpene Derivatives” OR “Derivatives, Tetraterpene” OR “Carotene” OR “Carotenes” OR “beta Carotene” OR “Vetoron” OR “BellaCarotin” OR “Betacarotene” OR “Carotene, beta” OR “Carotaben” OR “Provatene” OR “Solatene” OR “Max Caro” OR “MaxCaro” OR “Lycopene” OR “LYCOMATO” OR “LYC O MATO” OR “All trans Lycopene” OR “Pro Lycopene” OR “Prolycopene” OR “lutein” OR “gamma Lutein” OR “Lutein, gamma” OR “Lutein G” OR “Lutein F” OR “zeaxanthins” OR “Zeaxanthin” OR “Phenol” OR “Hydroxybenzene” OR “Carbolic Acid” OR “Carbol” OR “Phenol, Sodium Salt” OR “Phenolate, Sodium” OR “Sodium Phenolate” OR “Phenolate Sodium” OR “flavonoids“ OR “2 Phenyl Benzopyrans” OR “2 Phenyl Benzopyran” OR “2 Phenyl Chromene” OR “Flavonoid” OR “2 Phenyl Chromenes” OR “Bioflavonoid” OR “Bioflavonoids” OR “anthocyanins” OR “Leucoanthocyanidins” OR “Anthocyanin” OR “Anthocyanidin” OR “Anthocyanidins” OR “tea” OR “Tea, Black” OR “Black Teas” OR “Black Tea” OR “Teas, Black” OR “Green Tea” OR “Tea, Green” OR “Teas, Green” OR “Green Teas” OR “Curcumin” OR “Yellow, Turmeric” OR “Turmeric Yellow” OR “Phytosome, Curcumin” OR “Diferuloylmethane” OR “Curcumin Phytosome” OR “Mervia” OR “Resveratrol” OR “trans Resveratrol” OR “cis Resveratrol” OR “Resveratrol 3 sulfate” OR “SRT501” OR “SRT 501” OR “trans Resveratrol 3 O sulfate” OR “Dikvertin” OR “Quercetin” OR “Selenium” OR “Selenium 80” OR “Zinc” OR “Ubiquinone” OR “Coenzyme Q” OR “Ginkgo Extract” OR “Extract, Ginkgo” OR “Melatonin” OR “ergothioneine” OR “2 Thiol L histidine betaine” OR “Thioneine” OR “Thioctic Acid” OR “Neurium” OR “AlphaLipogamma” OR “Alpha Lipogamma” OR “Thiogamma oral” OR “Thioctacide T” OR “biomolipon” OR “biomo lipon” OR “Alpha Lipon Stada” OR “AlphaLipon Stada” OR “Alpha Lippon AL” OR “AlphaLippon AL” OR “alpha Liponsaure von ct” OR “alphaLiponsaure von ct” OR “Thioctacid” OR “Lipoic Acid” OR “alpha Lipoic Acid” OR “Thiogamma Injekt” OR “Injekt, Thiogamma” OR “alpha Vibolex” OR “alphaVibolex” OR “Tromlipon” OR “MTW Alphaliponsaure” OR “MTWAlphaliponsaure” OR “espa lipon” OR “espalipon” OR “Alphaflam” OR “PleomixAlpha” OR “Pleomix Alpha” OR “Azulipont” OR “alphaLiponaure Heumann” OR “alpha Liponaure Heumann” OR “Verla Lipon” OR “VerlaLipon” OR “PleomixAlpha N” OR “Pleomix Alpha N” OR “Fenint” OR “Juthiac” OR “duralipon” OR “Liponsaureratiopharm” OR “Liponsaure ratiopharm” OR “AlphaLiponsaure Sofotec” OR “Alpha Liponsaure Sofotec”)  #3 TS=((randomized controlled trial OR controlled clinical trial OR randomized OR placebo OR drug therapy OR randomly OR trial OR groups) NOT (animals NOT humans))  #4 #1 AND #2 AND #3 |
| Cochrane | #1 MeSH descriptor: [Antioxidants] explode all trees 6658  #2 MeSH descriptor: [Vitamin A] explode all trees 2610  #3 MeSH descriptor: [Ascorbic Acid] explode all trees 2837  #4 MeSH descriptor: [Tocopherols] explode all trees 889  #5 MeSH descriptor: [Carotenoids] explode all trees 4756  #6 MeSH descriptor: [beta Carotene] explode all trees 955  #7 MeSH descriptor: [Lycopene] explode all trees 319  #8 MeSH descriptor: [Luteinization] explode all trees 33  #9 MeSH descriptor: [Zeaxanthins] explode all trees 184  #10 MeSH descriptor: [Phenols] explode all trees 38512  #11 MeSH descriptor: [Flavonoids] explode all trees 3321  #12 MeSH descriptor: [Anthocyanins] explode all trees 232  #13 MeSH descriptor: [Tea] explode all trees 721  #14 MeSH descriptor: [Curcumin] explode all trees 731  #15 MeSH descriptor: [Resveratrol] explode all trees 428  #16 MeSH descriptor: [Quercetin] explode all trees 277  #17 MeSH descriptor: [Selenium] explode all trees 990  #18 MeSH descriptor: [Zinc] explode all trees 2082  #19 MeSH descriptor: [Ubiquinone] explode all trees 750  #20 MeSH descriptor: [Ginkgo Extract] explode all trees 4  #21 MeSH descriptor: [Melatonin] explode all trees 1763  #22 MeSH descriptor: [Thioctic Acid] explode all trees 458  #23 #1 OR #2 OR #3 OR #4 OR #5 OR #6 OR #7 OR #8 OR #9 OR #10 OR #11 OR #12 OR #13 OR #14 OR #15 OR #16 OR #17 OR #18 OR #19 OR #20 OR #21 OR #22 57975  #24 (‘Anti Oxidant’ OR ‘Anti-Oxidants’ OR ‘Anti-Oxidant’ OR ‘Antioxidant’ OR ‘Anti Oxidants’ OR ‘Activity, Antioxidant’ OR ‘Antioxidant Activity’ OR ‘Anti-Oxidant Effects’ OR ‘Anti Oxidant Effect’ OR ‘Antioxidant Effect’ OR ‘Anti Oxidant Effects’ OR ‘Anti-Oxidant Effect’ OR ‘Antioxidant Effects’ OR ‘Antioxidant, Endogenous’ OR ‘Antioxidants, Endogenous’ OR ‘Endogenous Antioxidants’ OR ‘Endogenous Antioxidant’ OR ‘All Trans Retinol’ OR ‘Retinol’ OR ‘Vitamin A1’ OR ‘quasol A’ OR ‘Magnorbin’ OR ‘Vitamin C’ OR ‘L Ascorbic Acid’ OR ‘Acid, Ascorbic’ OR ‘Ascorbate, Sodium’ OR ‘Sodium Ascorbate’ OR ‘Ascorbic Acid, Monosodium Salt’ OR ‘Magnesium di L Ascorbate’ OR ‘Magnesium Ascorbate’ OR ‘Magnesium Ascorbicum’ OR ‘Ascorbate, Magnesium’ OR ‘Ferrous Ascorbate’ OR ‘Ascorbate, Ferrous’ OR ‘Hybrin’ OR ‘Vitamin E, Vitagutt’ OR ‘Vitagutt Vitamin E’ OR ‘Mowivit Vitamin E’ OR ‘Vitamin E, Mowivit’ OR ‘Vitamin E EVI MIRALE’ OR ‘Vitamine E GNR’ OR ‘Vitamin E Dragees’ OR ‘E Vitamin E’ OR ‘Vitamin E mp’ OR ‘Vitamin E Suspension’ OR ‘Vitamin E, Togasan’ OR ‘Togasan Vitamin E’ OR ‘Vitamin E Natur’ OR ‘EUNOVA Vitamin E’ OR ‘Vitamin E AL’ OR ‘Vitamin E Sanum’ OR ‘Carotenoid’ OR ‘Tetraterpenes’ OR ‘Tetraterpene Derivatives’ OR ‘Derivatives, Tetraterpene’ OR ‘Carotene’ OR ‘Carotenes’ OR ‘Vetoron’ OR ‘BellaCarotin’ OR ‘Betacarotene’ OR ‘Carotene, beta’ OR ‘Carotaben’ OR ‘Provatene’ OR ‘Solatene’ OR ‘Max Caro’ OR ‘MaxCaro’ OR ‘LYCOMATO’ OR ‘LYC O MATO’ OR ‘All trans Lycopene’ OR ‘Pro Lycopene’ OR ‘Prolycopene’ OR ‘gamma Lutein’ OR ‘Lutein, gamma’ OR ‘Lutein G’ OR ‘Lutein F’ OR ‘Zeaxanthin’ OR ‘Hydroxybenzene’ OR ‘Carbolic Acid’ OR ‘Carbol’ OR ‘Phenol, Sodium Salt’ OR ‘Phenolate, Sodium’ OR ‘Sodium Phenolate’ OR ‘Phenolate Sodium’ OR ‘2 Phenyl Benzopyrans’ OR ‘2 Phenyl Benzopyran’ OR ‘2 Phenyl Chromene’ OR ‘Flavonoid’ OR ‘2 Phenyl Chromenes’ OR ‘Bioflavonoid’ OR ‘Bioflavonoids’ OR ‘Leucoanthocyanidins’ OR ‘Anthocyanin’ OR ‘Anthocyanidin’ OR ‘Anthocyanidins’ OR ‘Tea, Black’ OR ‘Black Teas’ OR ‘Black Tea’ OR ‘Teas, Black’ OR ‘Green Tea’ OR ‘Tea, Green’ OR ‘Teas, Green’ OR ‘Green Teas’ OR ‘Yellow, Turmeric’ OR ‘Turmeric Yellow’ OR ‘Phytosome, Curcumin’ OR ‘Diferuloylmethane’ OR ‘Curcumin Phytosome’ OR ‘Mervia’ OR ‘trans Resveratrol’ OR ‘cis Resveratrol’ OR ‘Resveratrol 3 sulfate’ OR ‘SRT501’ OR ‘SRT 501’ OR ‘trans Resveratrol 3 O sulfate’ OR ‘Dikvertin’ OR ‘Selenium 80’ OR ‘Coenzyme Q’ OR ‘Extract, Ginkgo’ OR ‘2 Thiol L histidine betaine’ OR ‘Thioneine’ OR ‘Neurium’ OR ‘AlphaLipogamma’ OR ‘Alpha Lipogamma’ OR ‘Thiogamma oral’ OR ‘Thioctacide T’ OR ‘biomolipon’ OR ‘biomo lipon’ OR ‘Alpha Lipon Stada’ OR ‘AlphaLipon Stada’ OR ‘Alpha Lippon AL’ OR ‘AlphaLippon AL’ OR ‘alpha Liponsaure von ct’ OR ‘alphaLiponsaure von ct’ OR ‘Thioctacid’ OR ‘Lipoic Acid’ OR ‘alpha Lipoic Acid’ OR ‘Thiogamma Injekt’ OR ‘Injekt, Thiogamma’ OR ‘alpha Vibolex’ OR ‘alphaVibolex’ OR ‘Tromlipon’ OR ‘MTW Alphaliponsaure’ OR ‘MTWAlphaliponsaure’ OR ‘espa lipon’ OR ‘espalipon’ OR ‘Alphaflam’ OR ‘PleomixAlpha’ OR ‘Pleomix Alpha’ OR ‘Azulipont’ OR ‘alphaLiponaure Heumann’ OR ‘alpha Liponaure Heumann’ OR ‘Verla Lipon’ OR ‘VerlaLipon’ OR ‘PleomixAlpha N’ OR ‘Pleomix Alpha N’ OR ‘Fenint’ OR ‘Juthiac’ OR ‘duralipon’ OR ‘Liponsaureratiopharm’ OR ‘Liponsaure ratiopharm’ OR ‘AlphaLiponsaure Sofotec’ OR ‘Alpha Liponsaure Sofotec’):ti,ab,kw 34583  #25 #23 OR #24 81021  #26 MeSH descriptor: [Glaucoma, Open-Angle] explode all trees 2369  #27 MeSH descriptor: [Low Tension Glaucoma] explode all trees 64  #28 (‘Normal Tension Glaucomas’ OR ‘ Normal Tension Glaucoma’ OR ‘ Glaucoma, Normal Tension’ OR ‘ Glaucoma, Low Tension’ OR ‘ Low Tension Glaucomas’ OR ‘Glaucoma, Secondary Open Angle’ OR ‘ Secondary Open Angle Glaucoma’ OR ‘ Open-Angle Glaucoma’ OR ‘ Open Angle Glaucoma’ OR ‘ Glaucoma, Pigmentary’ OR ‘ Simplex, Glaucoma’ OR ‘ Compensative Glaucoma’ OR ‘ Pigmentary Glaucoma’ OR ‘ Simple Glaucoma’ OR ‘ Glaucoma, Simple’ OR ‘ Glaucoma, Open Angle’ OR ‘ Open Angle Glaucomas’ OR ‘ Open-Angle Glaucomas’ OR ‘ Simplices, Glaucoma’ OR ‘ Compensated Glaucoma’ OR ‘ Glaucoma Simplex’ OR ‘ Glaucoma, Compensative’ OR ‘ Glaucoma, Compensated’ OR ‘ Chronic Primary Open Angle Glaucoma’ OR ‘ Glaucoma, Primary Open Angle’ OR ‘ Primary Open Angle Glaucoma’):ti,ab,kw 4574  #29 #26 OR #27 OR #28 4602  #30 #25 AND #29 121 |
| Embase | #1 'open angle glaucoma'/exp OR 'low tension glaucoma'/exp  #2 'glaucoma simplex':ti,ab,kw OR 'glaucoma, open angle':ti,ab,kw OR 'glaucoma, open-angle':ti,ab,kw OR 'open-angle glaucoma':ti,ab,kw OR 'primary open angle glaucoma':ti,ab,kw OR 'simple glaucoma':ti,ab,kw OR 'wide angle glaucoma':ti,ab,kw OR 'open angle glaucoma':ti,ab,kw OR 'glaucoma, low tension':ti,ab,kw OR 'normal tension glaucoma':ti,ab,kw OR 'low tension glaucoma':ti,ab,kw  #3 #1 OR #2 28142  #4 'antioxidant'/exp OR 'retinol'/exp OR 'ascorbic acid'/exp OR 'tocopherol'/exp OR 'carotenoid'/exp OR 'beta carotene'/exp OR 'lycopene'/exp OR 'xanthophyll'/exp OR 'astaxanthin'/exp OR 'zeaxanthin'/exp OR 'flavonoid'/exp OR 'anthocyanin'/exp OR 'tea'/exp OR 'curcumin'/exp OR 'resveratrol'/exp OR 'quercetin'/exp OR 'selenium'/exp OR 'zinc'/exp OR 'ubidecarenone'/exp OR 'ginkgo biloba extract'/exp OR 'melatonin'/exp OR 'thioneine'/exp OR 'thioctic acid'/exp  #5 'a 313':ti,ab,kw OR 'a fil':ti,ab,kw OR 'a mulsal':ti,ab,kw OR 'a mulsin':ti,ab,kw OR 'a mulsine':ti,ab,kw OR 'a sol':ti,ab,kw OR 'a vi pel':ti,ab,kw OR 'a vit':ti,ab,kw OR 'a vitadit':ti,ab,kw OR 'a vitamin':ti,ab,kw OR 'a vitan':ti,ab,kw OR 'a313':ti,ab,kw OR 'acon':ti,ab,kw OR 'acrisina':ti,ab,kw OR 'acrisine':ti,ab,kw OR 'actifral a':ti,ab,kw OR 'adatone':ti,ab,kw OR 'afaxin':ti,ab,kw OR 'afaxine':ti,ab,kw OR 'afilina':ti,ab,kw OR 'afiline':ti,ab,kw OR 'agiolan':ti,ab,kw OR 'alcovit a':ti,ab,kw OR 'alfa monovite':ti,ab,kw OR 'alfa sir':ti,ab,kw OR 'alfaergin':ti,ab,kw OR 'alfaergine':ti,ab,kw OR 'alfamin':ti,ab,kw OR 'alfamine':ti,ab,kw OR 'alfamonovit':ti,ab,kw OR 'alfasir':ti,ab,kw OR 'alfasole':ti,ab,kw OR 'alfasterolo':ti,ab,kw OR 'alfatar':ti,ab,kw OR 'alfavena':ti,ab,kw OR 'alfavene':ti,ab,kw OR 'alfavitina':ti,ab,kw OR 'alfavitine':ti,ab,kw OR 'alfene':ti,ab,kw OR 'all trans retinol':ti,ab,kw OR 'alphalin':ti,ab,kw OR 'alphaline':ti,ab,kw OR 'alphasterol':ti,ab,kw OR 'amulsal':ti,ab,kw OR 'amulsin':ti,ab,kw OR 'amulsine':ti,ab,kw OR 'amulvit':ti,ab,kw OR 'anatola':ti,ab,kw OR 'anatole':ti,ab,kw OR 'anavit':ti,ab,kw OR 'anti infective vitamin':ti,ab,kw OR 'antixerophthalmic vitamin':ti,ab,kw OR 'aoral':ti,ab,kw OR 'apexol':ti,ab,kw OR 'apostavit':ti,ab,kw OR 'arcavit a':ti,ab,kw OR 'asol':ti,ab,kw OR 'asteril':ti,ab,kw OR 'atav':ti,ab,kw OR 'aterapion':ti,ab,kw OR 'avibon':ti,ab,kw OR 'avibon theraplix':ti,ab,kw OR 'avimin':ti,ab,kw OR 'avimine':ti,ab,kw OR 'avipel':ti,ab,kw OR 'avipur':ti,ab,kw OR 'avit':ti,ab,kw OR 'avitabiol':ti,ab,kw OR 'avitadit':ti,ab,kw OR 'avital':ti,ab,kw OR 'avitaminum kolin':ti,ab,kw OR 'avitan':ti,ab,kw OR 'avitana':ti,ab,kw OR 'avitane':ti,ab,kw OR 'avite':ti,ab,kw OR 'avitil':ti,ab,kw OR 'avitina':ti,ab,kw OR 'avitol':ti,ab,kw OR 'avogina':ti,ab,kw OR 'avogine':ti,ab,kw OR 'avoleum':ti,ab,kw OR 'axerodina':ti,ab,kw OR 'axerodine':ti,ab,kw OR 'axerol':ti,ab,kw OR 'axerophthol':ti,ab,kw OR 'axerophthylium':ti,ab,kw OR 'bentavit a':ti,ab,kw OR 'bentavite a':ti,ab,kw OR 'bio tan':ti,ab,kw OR 'biosterol':ti,ab,kw OR 'biotan':ti,ab,kw OR 'chivibit a':ti,ab,kw OR 'cytobiase':ti,ab,kw OR 'dagravit a':ti,ab,kw OR 'davitamon a':ti,ab,kw OR 'difvitamin a':ti,ab,kw OR 'dohyfral a':ti,ab,kw OR 'elageno a':ti,ab,kw OR 'endo a':ti,ab,kw OR 'envit a':ti,ab,kw OR 'epiteliol':ti,ab,kw OR 'evitol zambeletti':ti,ab,kw OR 'fletase':ti,ab,kw OR 'gadeol':ti,ab,kw OR 'gadol':ti,ab,kw OR 'halivitan':ti,ab,kw OR 'halivitane':ti,ab,kw OR 'homagenets aoral':ti,ab,kw OR 'hydrosol':ti,ab,kw OR 'ido a':ti,ab,kw OR 'ido a 50':ti,ab,kw OR 'idratene':ti,ab,kw OR 'inovitan a':ti,ab,kw OR 'lord factor':ti,ab,kw OR 'meditalfa':ti,ab,kw OR 'mulsal a':ti,ab,kw OR 'multamine':ti,ab,kw OR 'nio a let':ti,ab,kw OR 'oleovit a':ti,ab,kw OR 'oleovitamin a':ti,ab,kw OR 'ophthalamin':ti,ab,kw OR 'panvita':ti,ab,kw OR 'plivit a':ti,ab,kw OR 'prepalin':ti,ab,kw OR 'prepaline':ti,ab,kw OR 'preparato a':ti,ab,kw OR 'primavit':ti,ab,kw OR 'quotivit':ti,ab,kw OR 'retinol alcohol':ti,ab,kw OR 'retinyl alcohol':ti,ab,kw OR 'ro a vit':ti,ab,kw OR 'super a':ti,ab,kw OR 'testavol':ti,ab,kw OR 'ucemine a':ti,ab,kw OR 'vaconex':ti,ab,kw OR 'vaflol':ti,ab,kw OR 'veroftal':ti,ab,kw OR 'vi alpha':ti,ab,kw OR 'vi dom a':ti,ab,kw OR 'viadenin':ti,ab,kw OR 'vialpha':ti,ab,kw OR 'viatate':ti,ab,kw OR 'vidoma':ti,ab,kw OR 'vitadone':ti,ab,kw OR 'vitadral':ti,ab,kw OR 'vitalen a':ti,ab,kw OR 'vitalfa':ti,ab,kw OR 'vitama':ti,ab,kw OR 'vitamin a':ti,ab,kw OR 'vitamin a alcohol':ti,ab,kw OR 'vitamin a1':ti,ab,kw OR 'vitaplex a':ti,ab,kw OR 'vitapur a':ti,ab,kw OR 'vitasan a':ti,ab,kw OR 'vitavel a':ti,ab,kw OR 'vitpex':ti,ab,kw OR 'vogan':ti,ab,kw OR 'vogan neu':ti,ab,kw OR 'wandervit a':ti,ab,kw OR 'xerophthol':ti,ab,kw OR 'retinol':ti,ab,kw OR 'tocoferol':ti,ab,kw OR 'tocopherols':ti,ab,kw OR 'tocopherol':ti,ab,kw OR 'carotenoid pigment':ti,ab,kw OR 'carotenoids':ti,ab,kw OR 'carotinoid':ti,ab,kw OR 'carotenoid':ti,ab,kw OR 'beta carotin':ti,ab,kw OR 'betacarotene':ti,ab,kw OR 'betatene':ti,ab,kw OR 'carotaben':ti,ab,kw OR 'lurotin':ti,ab,kw OR 'natural betacarotene':ti,ab,kw OR 'solatene':ti,ab,kw OR 'solvin':ti,ab,kw OR 'trans beta carotene':ti,ab,kw OR 'beta carotene':ti,ab,kw OR 'lycopene':ti,ab,kw OR 'cis xantofyl':ti,ab,kw OR 'lutein':ti,ab,kw OR 'luteine':ti,ab,kw OR 'xanthophyl':ti,ab,kw OR 'xanthophylls':ti,ab,kw OR 'xantofyl':ti,ab,kw OR 'xantophyl':ti,ab,kw OR 'xanthophyll':ti,ab,kw OR 'astaxanthine':ti,ab,kw OR 'ovoester':ti,ab,kw OR 'astaxanthin':ti,ab,kw OR 'zeaxanthins':ti,ab,kw OR 'zeaxantin':ti,ab,kw OR 'zeaxanthin':ti,ab,kw OR 'flavonoid derivative':ti,ab,kw OR 'flavonoids':ti,ab,kw OR 'flavonoid':ti,ab,kw OR 'anthocyanine':ti,ab,kw OR 'anthocyanins':ti,ab,kw OR 'anthocyanin':ti,ab,kw OR 'black tea':ti,ab,kw OR 'green tea':ti,ab,kw OR 'nb tea':ti,ab,kw OR 'pu erh tea':ti,ab,kw OR 'puer tea':ti,ab,kw OR 'puerh tea':ti,ab,kw OR 'tea extract':ti,ab,kw OR 'tea infusion':ti,ab,kw OR 'tea infusions':ti,ab,kw OR 'white tea':ti,ab,kw OR 'tea':ti,ab,kw OR 'curcumine':ti,ab,kw OR 'diferuloylmethane':ti,ab,kw OR 'nanocurc':ti,ab,kw OR 'turmeric yellow':ti,ab,kw OR 'curcumin':ti,ab,kw OR 'srt 501':ti,ab,kw OR 'srt501':ti,ab,kw OR 'trans resveratrol':ti,ab,kw OR 'resveratrol':ti,ab,kw OR 'ascorbic acid plus quercetin':ti,ab,kw OR 'flavin':ti,ab,kw OR 'hippuroflavin':ti,ab,kw OR 'meletin':ti,ab,kw OR 'meltin':ti,ab,kw OR 'quercetine':ti,ab,kw OR 'quercetol':ti,ab,kw OR 'quercetole':ti,ab,kw OR 'quercitin':ti,ab,kw OR 'quertine':ti,ab,kw OR 'sophoretin':ti,ab,kw OR 'quercetin':ti,ab,kw OR '80se':ti,ab,kw OR 'novamed selen':ti,ab,kw OR 'radioactive selenium':ti,ab,kw OR 'radioselenium':ti,ab,kw OR 'se':ti,ab,kw OR 'selenicum':ti,ab,kw OR 'selenium radioisotopes':ti,ab,kw OR 'selenium':ti,ab,kw OR '64zn':ti,ab,kw OR 'zinc 64':ti,ab,kw OR 'zinc chelate':ti,ab,kw OR 'zinc content':ti,ab,kw OR 'zinc dust':ti,ab,kw OR 'zinc isotopes':ti,ab,kw OR 'zinc radioisotopes':ti,ab,kw OR 'zinc retention':ti,ab,kw OR 'zincum':ti,ab,kw OR 'zn':ti,ab,kw OR 'zn 64':ti,ab,kw OR 'zinc':ti,ab,kw OR 'caomet':ti,ab,kw OR 'coenzyme 910':ti,ab,kw OR 'coenzyme q 10':ti,ab,kw OR 'coenzyme q10':ti,ab,kw OR 'decorenone':ti,ab,kw OR 'mitocor':ti,ab,kw OR 'neuquinone':ti,ab,kw OR 'quinone q 10':ti,ab,kw OR 'ubimaior':ti,ab,kw OR 'ubiquinone 10':ti,ab,kw OR 'ubiquinone 50':ti,ab,kw OR 'ubiten':ti,ab,kw OR 'ubidecarenone':ti,ab,kw OR 'egb 761':ti,ab,kw OR 'egb761':ti,ab,kw OR 'gingko biloba extract':ti,ab,kw OR 'ginkgo extract':ti,ab,kw OR 'ginkgold':ti,ab,kw OR 'ginkgopower':ti,ab,kw OR 'ginkobene':ti,ab,kw OR 'ginkor':ti,ab,kw OR 'kaveri':ti,ab,kw OR 'kaveri forte':ti,ab,kw OR 'li 1370':ti,ab,kw OR 'rokan':ti,ab,kw OR 'superginkgo':ti,ab,kw OR 'tanakan':ti,ab,kw OR 'tanakene':ti,ab,kw OR 'tebonin':ti,ab,kw OR 'ginkgo biloba extract':ti,ab,kw OR '5 methoxy n acetyltryptamine':ti,ab,kw OR 'adaflex':ti,ab,kw OR 'apl 510':ti,ab,kw OR 'apl510':ti,ab,kw OR 'aritonin':ti,ab,kw OR 'bci 049':ti,ab,kw OR 'bci049':ti,ab,kw OR 'celton xl':ti,ab,kw OR 'ceyestaeusom':ti,ab,kw OR 'ceyesto':ti,ab,kw OR 'circadin':ti,ab,kw OR 'civasta':ti,ab,kw OR 'clemenos xl':ti,ab,kw OR 'eusom':ti,ab,kw OR 'jan 13004':ti,ab,kw OR 'jan13004':ti,ab,kw OR 'ki 1001':ti,ab,kw OR 'ki1001':ti,ab,kw OR 'lestinora':ti,ab,kw OR 'mallozen':ti,ab,kw OR 'mecastrin':ti,ab,kw OR 'melabiorytm':ti,ab,kw OR 'melatal':ti,ab,kw OR 'melatan':ti,ab,kw OR 'melatol':ti,ab,kw OR 'melatonina':ti,ab,kw OR 'melatonine':ti,ab,kw OR 'melatonite':ti,ab,kw OR 'mellaras':ti,ab,kw OR 'mellozzan':ti,ab,kw OR 'melovine':ti,ab,kw OR 'mucomel':ti,ab,kw OR 'n acetyl 5 methoxytryptamine':ti,ab,kw OR 'noxarem':ti,ab,kw OR 'orlogin':ti,ab,kw OR 'sental':ti,ab,kw OR 'slenyto':ti,ab,kw OR 'sleplag':ti,ab,kw OR 'sloremina':ti,ab,kw OR 'sp 13004':ti,ab,kw OR 'sp13004':ti,ab,kw OR 'syncrodin':ti,ab,kw OR 'waferest':ti,ab,kw OR 'melatonin':ti,ab,kw OR 'ergothioneine':ti,ab,kw OR 'ergothionone':ti,ab,kw OR 'sympectothion':ti,ab,kw OR 'thiasine':ti,ab,kw OR 'thiohistidine trimethylbetaine':ti,ab,kw OR 'thiolhistidine betaine':ti,ab,kw OR 'thiozone':ti,ab,kw OR 'thioneine':ti,ab,kw OR 'alpha lipoic acid':ti,ab,kw OR 'alpha liponic acid':ti,ab,kw OR 'berlithione':ti,ab,kw OR 'biletan':ti,ab,kw OR 'dextro levo alpha lipoic acid':ti,ab,kw OR 'dl alpha lipoic acid':ti,ab,kw OR 'heparlipon':ti,ab,kw OR 'lipoate':ti,ab,kw OR 'lipoic acid':ti,ab,kw OR 'liponic acid':ti,ab,kw OR 'neurothioct':ti,ab,kw OR 'protogen':ti,ab,kw OR 'protogen a':ti,ab,kw OR 'thioctacid':ti,ab,kw OR 'thioctan':ti,ab,kw OR 'thiogamma':ti,ab,kw OR 'thiotocid':ti,ab,kw OR 'tioctan':ti,ab,kw OR 'tioctic acid':ti,ab,kw OR 'tioctidasi':ti,ab,kw OR 'thioctic acid':ti,ab,kw  #6 #4OR#5  #7 'crossover procedure':de OR 'double-blind procedure':de OR 'randomized controlled trial':de OR 'single-blind procedure':de OR random*:de,ab,ti OR factorial*:de,ab,ti OR crossover*:de,ab,ti OR ((cross NEXT/1 over*):de,ab,ti) OR placebo*:de,ab,ti OR ((doubl* NEAR/1 blind*):de,ab,ti) OR ((singl* NEAR/1 blind*):de,ab,ti) OR assign*:de,ab,ti OR allocat*:de,ab,ti OR volunteer*:de,ab,ti  #8 #3 AND #6 AND #7 |
